# Mitochondrial damage and IL-1β production in monocytes caused by *Neospora caninum* infection are mediated by dense granule protein 7 and prohibitins

**DOI:** 10.3389/fimmu.2025.1408992

**Published:** 2025-11-20

**Authors:** Yu Chen, Naomi Shimoda, Coh-ichi Nihei, Ryuichi Sawa, Yuko Takahashi, Mitsuhiro Nishigori, Shu Nakamura, Jingwei Liu, Takumi Koshiba, Nanako Ushio-Watanabe, Yoshifumi Nishikawa

**Affiliations:** 1 National Research Center for Protozoan Diseases, Obihiro University of Agriculture and Veterinary Medicine, Obihiro, Japan; 2 Institute of Microbial Chemistry (BIKAKEN), Tokyo, Japan; 3 Department of Chemistry, Faculty of Science, Fukuoka University, Fukuoka, Japan

**Keywords:** NcGRA7, NLRP3 inflammasome, mitochondria, PHB1, PHB2, monocyte

## Abstract

**Introduction:**

The intracellular proliferation of *Neospora caninum* tachyzoites and the host immune response against infection are key steps in the pathogenesis of neosporosis. However, the molecules responsible for the activation of the inflammasome induced by *N. caninum* infection have not been identified.

**Methods:**

In the infection of human monocytic cell line THP-1 cells with the *N. caninum* dense granule protein 7 knock-out (NcGRA7KO) parasite and the parental strain Nc1, production of IL-1β and TNF-α, phosphorylated NF-κB p65 were measured. LC‒MS/MS analysis of NcGRA7 immunoprecipitates, mitochondrial fractionation and proteolysis assays were also performed.

**Results:**

In the infection of THP-1 cells with the NcGRA7KO parasite, decreased IL-1β release was observed compared with Nc1 infection. Transfection with plasmids for reconstitution of the NLRP3 inflammasome enhanced IL-1b production via additional transfection with NcGRA7 cDNA. NLRP3 inhibitor, CASP1 inhibitor, and NF-κB inhibitor significantly suppressed IL-1β production induced by Nc-1 infection, whereas no inhibitory effect was observed in NcGRA7KO-infected cells. Furthermore, treatment with any of the inhibitors led to a reduction in TNF-α production in both Nc-1- and NcGRA7KO-infected cells. Western blot analysis of phosphorylated NF-κB p65 demonstrated that *N. caninum* infection induced NF-κB p65 phosphorylation; however, no significant difference was observed between Nc1-infected and NcGRA7KO-infected cells. Infection of the THP-1 cells with NcGRA7KO parasites decreased the host mitochondrial damage and apoptosis in THP-1 cells compared to infection with Nc1, suggesting that NcGRA7 plays a crucial role in the pathogenesis of *N. caninum*. Furthermore, LC‒MS/MS analysis of NcGRA7 immunoprecipitates identified NcGRA7-binding proteins in host cells that localize to host mitochondria. Additionally, mitochondrial fractionation and proteolysis assays using proteinase K showed the distribution of NcGRA7 from the inner mitochondrial membrane to the matrix of host mitochondria. Interestingly, NcGRA7 formed a complex with the prohibitins PHB1 and PHB2. Using inhibitors of PHB1 or transfecting *N. caninum*-infected THP-1 cells with PHB1 siRNA significantly decreased IL-1β production, but not TNF-α.

**Discussion:**

These findings indicate that NcGRA7 does not directly modulate NF-κB activation but likely enhances the production of IL-1β and TNF-α through post-transcriptional mechanisms, thereby contributing to the upregulation of NLRP3 expression. Moreover, our results suggest that NcGRA7 influences both the NF-κB and inflammasome pathways through its interaction with host cell prohibitins. Elucidating the roles of PHB1 and NcGRA7 will provide new insights into the host–parasite interactions underlying *N. caninum* pathogenesis.

## Introduction

*Neospora caninum* is an obligate intracellular protozoan parasite of the phylum Apicomplexa; its intermediate hosts include a wide range of domestic and wild animals, and the definitive hosts of *N. caninum* are dogs and other canids (1). Neosporosis caused by *N. caninum* infection often induces abortion, especially in cattle, as well as stillbirth and the birth of weak calves, leading to global economic losses in both the dairy and beef industries (2, 3). Because there is no treatment or vaccine available against neosporosis in cattle, effective drugs and vaccines are still urgently needed to treat this disease (4).

The intracellular proliferation of *N. caninum* tachyzoites and the host immune response against infection are key steps in the pathogenesis of neosporosis (4, 5). The innate immune system is the first line of defense against *N. caninum* infection because it can control initial parasite replication and subsequently mediate appropriate adaptive immunity via a Th1-type protective immune response mediated by the production of the proinflammatory cytokines interferon-gamma (IFN-γ) and interleukin (IL)-12p40 (4, 6), resulting in parasite clearance or establishment of chronic infection.

Pattern recognition receptors (PRRs) of innate immune cells, such as macrophages, can sense microbes by recognizing pathogen-associated molecular patterns (PAMPs) and danger-associated molecular patterns (DAMPs) via Toll-like receptors (TLRs). In the case of *N. caninum* infection, TLR2 and TLR3 participate in the initial recognition of the parasites (7–9). Additionally, nucleotide oligomerization domain (Nod)-like receptors (NLRs), another group of PRRs, play important roles in the host response to intracellular parasites (10). The NLR can be defined by a tripartite structure (11), and two groups might be formed from these structures. One of these pathways mediates NF-κB activation via NOD1 and NOD2 (12). The other senses multiplying PAMPs and DAMPs, triggering the multiprotein platform known as the inflammasome to initiate host defense (13).

The nucleotide-binding domain leucine-rich repeat and pyrin domain containing receptor 3 (NLRP3) inflammasome is a cytosolic complex that functions as a sensor protein that belongs to one member of the NLR family. Several stimuli can trigger the activation of this complex, such as extracellular ATP, nigericin, and uric acid crystals (14) and bacterial pathogens, fungal pathogens, viruses, and parasites (11). When the NLRP3 inflammasome is activated by multiple agonists, including PAMPs or DAMPs, it interacts with the pyrin domain of apoptosis-associated speck-like protein containing a caspase recruitment domain (ASC), and the caspase-recruitment domain (CARD) of ASC subsequently binds the CARD domain of caspase-1, thereby forming the NLRP3 inflammasome (10, 15–17). This subsequently leads to the assembly of the protein complex and the cleavage of pro-IL-18 and pro-IL-1β by caspase-1 into their mature forms, allowing their release (18). On the other hand, activated caspase-1 promotes an inflammatory form of cell death called pyroptosis by cleaving the cytosolic substrate gasdermin D (GSDMD) (19). Moreover, the gasdermin D N-terminus integrates into the plasma membrane, forming pores and leading to the destabilization of cell membrane integrity (20). However, inflammasome activation does not consistently lead to pyroptotic cell death, for example, *Toxoplasma gondii*, which is closely related intracellular protozoan parasite with *N. caninum*, infection of primary human monocytes requires the NLRP3 inflammasome and caspase-1 but is independent of gasdermin D and pyroptosis (21). Also, caspase-8 regulates IL-1β secretion via a mechanism independent of gasdermin in *T. gondii*-infected human monocytes (22).

A previous study showed that NLRP1 encodes an inflammasome sensor for *T. gondii* that controls macrophage sensitivity to pyroptosis (23). In rat macrophages infected with *T. gondii*, knockdown of Nlrp1 resulted in increased parasite replication and protection from cell death (24). Mitochondria, which are important organelles for cytotoxicity, oxidative stress, and apoptosis, are closely associated with the activation of the NLRP3 inflammasome (25, 26). NLRP3 also plays an important role in limiting parasite replication for host resistance to toxoplasmosis (27). *T. gondii* also activates the NLRP12 inflammasome pathway (28). However, little is known about the inflammasome reactions involved in *N. caninum* infections, while NLRP3 inflammasome activation in murine macrophages caused by *N. caninum* infection contributes to the host response to control these parasites (29).

In *N. caninum*-infected bovine macrophages, activation of caspase-1 is mediated by the inflammasome, leading to caspase-1-dependent apoptosis (30). *N. caninum* infection induces the release of IL-1β and IL-18, cleaved caspase-1, and cell death in mouse bone marrow-derived macrophages, while infection of mice deficient in NLRP3, ASC, and caspase-1/11 leads to a decrease in IL-18 production and an increase in IFN-γ in the serum (31). During *N. caninum* infection, ASC, Caspase-1, NLRP3, and NLRC4 are necessary for inflammasome activation. In bone marrow-derived macrophages and mice, reactive oxygen species (ROS) production and inflammasome assembly via NF-κB activation lead to the restriction of *N. caninum* replication (32). Additionally, *N. caninum*-induced NLRP3 inflammasome activation via the NADPH-dependent ROS-mediated pathway has been confirmed (33).

Taken together, these findings indicate that the *N. caninum*-induced inflammasome plays a crucial role in the pathogenesis of neosporosis. However, the molecules responsible for the activation of the *N. caninum* inflammasome have not been identified, and the molecular mechanism affecting inflammasome activation remains to be further investigated. In this study, we found that *N. caninum* dense granule protein 7 (NcGRA7) contributed to the NF-κB and inflammasome pathways through its interaction with host cell prohibitins. This is the first study to identify that *N. caninum* molecules participate in inflammasome activation and are important for further understanding host defense against intercellular parasites.

## Materials and methods

### Ethics statement

We did not use experimental animals in this study.

### Parasite and cell culture

The *N. caninum* (Nc1 strain), *NcGRA6*-knockout, *NcGRA14*-knockout *NcGRA7*-knockout and *NcGRA7*-complemented parasites (34) were maintained in African green monkey kidney epithelial cells (Vero cells) cultured in Eagle’s minimum essential medium (EMEM; Sigma, St. Louis, MO, USA) supplemented with 8% heat-inactivated fetal bovine serum (FBS). *NcGRA7*-complemented parasites was generated by inserting *NcGRA7* gene fused with FLAG tag under *Toxoplasma* GRA1 promoter into the *Neospora* uracil phosphoribosyl transferase (NcUPRT) gene of the *NcGRA7*-knockout line.

Human monocytic leukemia cell line, THP-1 cells, were cultured in Roswell Park Memorial Institute (RPMI) 1640 medium (Sigma) supplemented with 10% heat-inactivated FBS. 293T cells and HFFs were cultured in Dulbecco’s modified Eagle’s medium (Sigma) supplemented with 10% heat-inactivated FBS.

For the purification of tachyzoites, the parasite cultures were washed with each medium, scraped, suspended in the medium and passed through a 27-gauge needle and a 5.0 μm pore filter (Millipore, Bedford, MA, USA). After centrifugation, the tachyzoites were resuspended in the medium as purified parasites.

### Reagents

MCC950 (an inhibitor of NLRP3 inflammasome activation by the inhibition of IL-1β release; Cayman Chemical), VX765 (an inhibitor of NLRP3 inflammasome activation by the inhibition of caspase 1 and IL-1β cleavage and release; LKT Laboratories), SN50 (an inhibitor of the nuclear transcription factor κB; Selleck, Houston, TX, USA), a carbonyl cyanide m-chlorophenyl hydrazone (CCCP, a uncoupling agent for oxidative phosphorylation that inhibits mitochondrial function; FUJFILM Wako Pure Chemical Corporation), rocaglamid A (Cayman, Ann Arbor, MI, USA) with biochemical research grades were used in this study.

### Plasmids

The p3XFLAG-CMV-14 plasmid (Sigma–Aldrich) encoding the C-terminal FLAG-tagged NcGRA7 (p3XFLAG-NcGRA7) was described previously (34). Plasmids encoding NLRP3, ASC, procaspase-1, and pro-IL-1β were kindly gifted from Dr. Takeshi Ichinohe (The University of Tokyo) (35).

### Cytokine enzyme-linked immunosorbent assay

The culture supernatants were collected to measure the IL-1β and TNF-α levels via ELISA kits (Human OptEIA ELISA Set, BD Biosciences, San Jose, CA, USA) according to the manufacturer’s instructions. The cytokine concentrations were calculated from curves generated from cytokine standards analyzed on the same plates.

### Measurement of mitochondrial membrane potential

JC-1 is widely used for observing mitochondrial membrane potential and shows changes in fluorescence characteristics from green (530 nm) to red (590 nm) depending on the mitochondrial membrane potential. For the JC-1 assay, cells were stained using a JC-1 MitoMP detection kit (Dojindo Laboratories, Kumamoto, Japan) according to the manufacturer’s instructions.

### Detection of apoptosis

Annexin V staining precedes the loss of membrane integrity, which accompanies the latest stages of cell death resulting from either apoptotic or necrotic processes. Used in conjunction with a vital dye such as 7-Amino-Actinomycin (7-AAD), cells that are considered viable are PE Annexin V and 7-AAD negative; cells that are in early apoptosis are PE Annexin V positive and 7-AAD negative; and cells that are in late apoptosis or already dead are both PE Annexin V and 7-AAD positive. For measurement of early apoptosis, cells were stained using a PE Annexin V Apoptosis Detection Kit I (BD Pharmingen, San Diego, CA, USA) according to the manufacturer’s instructions.

### LC–MS/MS

For LC–MS/MS analysis using immunoprecipitates, 293T cells at 80% confluence were transiently transfected with the p3XFLAG-NcGRA7 or empty plasmid (mock) (12 μg each) in a 10 cm culture dish using FuGENE HD (Promega). The next day, the cells were lysed with 1 mL of lysis buffer containing 50 mM Tris-HCl (pH 7.4), 150 mM NaCl, 1% NP-40, and protease inhibitor cocktail (Complete Mini, Roche), and the clarified supernatants were incubated for 16 h at 4°C with 20 μL of ANTI-FLAG M2 Affinity Gel (Sigma). After washing 3 times with IP buffer containing 10 mM Tris-HCl (pH 7.4), 150 mM NaCl and 0.5% NP-40, the immunoprecipitates were resolved via 10% SDS–PAGE.

The specific bands observed in the immunoprecipitated NcGRA7-FLAG were subjected to liquid chromatography-tandem mass spectrometry (LC–MS/MS) analysis. For protein identification, the eluted proteins were separated via SDS–PAGE and stained with silverstain, after which the gel regions containing the proteins of interest were excised. Proteins in the excised gel blocks were destained, reduced, alkylated at the cysteine residues, and digested with modified trypsin. Trypsin-digested peptides were extracted from the gel blocks and subjected to mass spectrometric analysis. LC–MS/MS was performed on a Paradigm MS2 HPLC (Michrom BioResources) and an LTQ linear ion trap mass spectrometer with a Nanospray ion source housing (Thermo Fisher Scientific). Peptides were separated through an L-column2 C18 (0.1 × 50 mm, 3 µm particle size, CERI, Tokyo) using a linear gradient (0.2% acetonitrile/B. 90% acetonitrile in 0.1% formic acid, 5–40% acetonitrile 2%/min) at a flow rate of 0.5 µl/min. Mass spectrometric data were searched against the IPI_human (91,464 sequences; 36,355,611 residues) database using MASCOT software ver. 2.8 (Matrix Science). The MASCOT score (target FDR/override significance threshold, 1%), sequence coverage, total number of mass-matched peptides, accession number of MS, IPI_human-database, and emPAI are shown. The emPAI (exponentially modified protein abundance index), which offers approximate and relative quantification of proteins in a mixture, was calculated on two parameters, the number of experimentally observed peptides, as described by Ishihama et al. (2005) (36).

### Antibodies

Anti-FLAG M2 mouse monoclonal antibody (Sigma–Aldrich), anti-XPOT (Exportin-T) rabbit antibody (A303-972A, Bethyl, Montgomery, TX, USA), anti-XPO1(CRM1) rabbit antibody (A300-469A, Bethyl), anti-SLC25A13 rabbit antibody (GTX109001, GeneTex, Irvine, CA, USA), anti-DNAJA1 rabbit antibody (A304-516A, Bethyl), anti-UQCRC2 rabbit antibody (GTX114873, GeneTex), anti-PHB1 rabbit antibody (GTX101105, GeneTex), anti-CoxIV rabbit monoclonal antibody (3E11, Cell Signaling Technology, Danvers, MA, USA), anti-GAPDH rabbit monoclonal antibody (14C10, Cell Signaling Technology), anti-Mfn1 rabbit antibody (13798-1-AP, Proteintech, Rosemont, IL, USA), anti-AIF mouse monoclonal antibody (sc-13116, Santa Cruz Biotechnology, Dallas, TX, USA), anti-mtHSP70 mouse monoclonal antibody (JG1, Invitrogen, Waltham, MA, USA), anti-VDAC1 rabbit antibody (4866, Cell Signaling Technology), anti-PHB2 mouse monoclonal antibody (sc-133094, Santa Cruz Biotechnology), anti-Histone H3 rabbit antibody (9715S, Cell Signaling Technology), anti-phospho-NF-κB p65 rabbit monoclonal antibody (93H1, Cell Signaling Technology) and anti-NFκB p65 rabbit polyclonal antibody (sc-109, Santa Cruz Biotechnology) were used for western blot and indirect fluorescent antibody test (IFAT) in this study. To detect *N. caninum* proteins, an anti-NcSRS2 mouse monoclonal antibody (clone 1B8) (37), anti-NcGRA7 mouse serum and anti-NcGRA7 rabbit antibody (34) were used.

### Immunoprecipitation and western blotting

HFFs at 80% confluence were infected with 5×10^6^ purified parasites (Nc1- and NcGRA7-complemented parasites) in a 10 cm culture dish. After 40 hr, the cells were lysed with 1 mL of lysis buffer, and the clarified supernatants were incubated for 16 h at 4°C with 20 μL of ANTI-FLAG M2 Affinity Gel (Sigma) followed by washing 3 times with IP buffer. The immunoprecipitates of the plasmid-transfected cells and the parasite-infected cells were resolved by 10-12% SDS–PAGE. and immunoblotted with antibodies against FLAG-tagged protein or the indicated proteins.

### IFAT

For mitochondrial staining, the cells were incubated with 200 nM MitoTracker^®^ Red CMXRos (Thermo Fisher Scientific, Waltham, MA, USA) for 30 min before fixation. Coverslips with cells were collected at the indicated time points, washed twice with PBS and then fixed with 2% paraformaldehyde in PBS. After washing twice with PBS, the cells were permeabilized with 0.3% Triton X-100 in PBS for 5 min at room temperature. After washing, the coverslips were incubated with 3% bovine serum albumin (BSA) in PBS at room temperature for 30 min, after which the sections were subjected to antibody staining and nuclear staining with Hoechst 33342 (Sigma–Aldrich). Observation was performed with a Leica Microsystems THUNDER Imaging System.

### Immune-electron microscopy

Vero cells infected with *N. caninum* (Nc1 strain) at 48 h postinfection were fixed with 4% paraformaldehyde (FUJIFILM Wako, Tokyo, Japan) for 1 h at room temperature and then washed with 0.1 M phosphate buffer three times. The samples were dehydrated through a series of alcohol series ranging from 70-100%, 100% alcohol was gradually changed with LR white resin (Nisshin EM, Tokyo, Japan), and the samples were incubated with 100% LR white resin overnight at 4°C. The samples were embedded in 100% LR white resin in a gelatin capsule (Nisshin EM) and incubated for 24 h at 50°C. Ultrathin sections were collected on carbon-coated nickel grids (ADD Deplen, Tokyo, Japan). After incubating with blocking buffer, 4% BSA and 0.1% sodium azide in PBS, the grids were incubated with dilutions of anti-NcGRA7 rabbit antibody or normal rabbit serum for 2 h at room temperature. After washing with PBS seven times, the grids were incubated with dilutions of gold (10 nm)-conjugated anti-rabbit IgG goat antibody (CytodiagnoSitc, Burlington, Canada) for 3 h at room temperature. After washing with PBS six times, the sections were fixed with 1% osmium tetroxide and stained with uranyl acetate and lead citrate. Finally, the sections were observed with a HITACHI HT7700 transmission electron microscope (Hitachi High-Tech Corporation, Tokyo, Japan).

### Mitochondrial fractionation and proteolysis

Mitochondrial fractionation was performed using mitochondria/cytosol fractionation kit as per manufacturer’s instructions (Enzo Life Sciences, NY, USA). The fractions of NP, Mt and Cyto were resolved in 100 μl of SDS–PAGE sample buffer (62.5 mM Tris-HCl (pH 6.8), 0.017% SDS, 5% glycerol, 5% 2-mercaptoethanol, and 0.005% bromophenol blue) for NP and Mt and 100 μl of SDS–PAGE sample buffer supplemented with 50 mM Tris-acetate (pH 7.5), 8 M urea, and 0.1% SDS for Cyto and subjected to SDS–PAGE followed by western blotting with the indicated antibodies.

Proteolysis assays using proteinase K were performed as previously described (38) with slight modifications. Briefly, cells in 10 cm cell culture dishes were washed twice with HBSS (Gibco), scraped off the culture plate, and lysed in 1 mL of homogenization buffer (20 mM HEPES [pH 7.5], 70 mM sucrose, and 220 mM mannitol) by 30 strokes in a Dounce homogenizer. The homogenate was centrifuged at 1,000×g for 5 min to precipitate the nuclei, and the resulting supernatant (300 μl each, 3 tubes) was further centrifuged at 14,000×g for 10 min (4°C) to precipitate the crude mitochondrial fraction. For the proteinase K resistance assay, the isolated mitochondrial pellet was resuspended in 200 μl of buffer, homogenization buffer, 100 μg/mL proteinase K in homogenization buffer or 100 μg/mL proteinase K in hypotonic swelling buffer (20 mM HEPES-KOH, pH 7.5). The samples were incubated for 15 min (on ice) and further centrifuged at 14,000×g for 15 min (4°C) to precipitate the crude mitochondrial fraction. The precipitates were resolved in 60 μl of 5 mM PMSF in SDS–PAGE sample buffer and subjected to western blotting with the indicated antibodies.

### RNA interference

For RNA interference knockdown experiments, Silencer™ Select Negative Control No. 1 siRNA (Thermo Fisher Scientific), PHB1 Sense siRNA sequence #2 (CGUGGGUACAGAAACCAAUtt) (Thermo Fisher Scientific) were used as negative controls and PHB1 knockdown, respectively. THP-1 cells were transfected with 30 pmol of siRNA using the SG Cell Line 4D-Nucleofector™ X Kit L (catalog #: V4XC-3024; Lonza, Basel, Switzerland) following the manufacturer’s protocols. At 20 h after transfection, the siRNA-treated cells were infected with *N. caninum* and subjected to functional assays. The protein levels after siRNA treatment were analyzed via western blotting. Band intensity was quantified using ImageJ software developed by the US National Institutes of Health.

### Statistical analysis

The data are expressed as the mean ± SD of 3–4 replicates in one representative experiment (each experiment was repeated two to four times). The various assay conditions were evaluated with analysis of variance (ANOVA) followed by Tukey’s multiple comparisons test. A *P* value (* *P* < 0.05, ** *P* < 0.01, *** *P* < 0.001, **** *P* < 0.0001) was considered to indicate statistical significance.

## Results

### Effects of NcGRA7 on production of IL-1β and TNF-α

**Figure 1:**
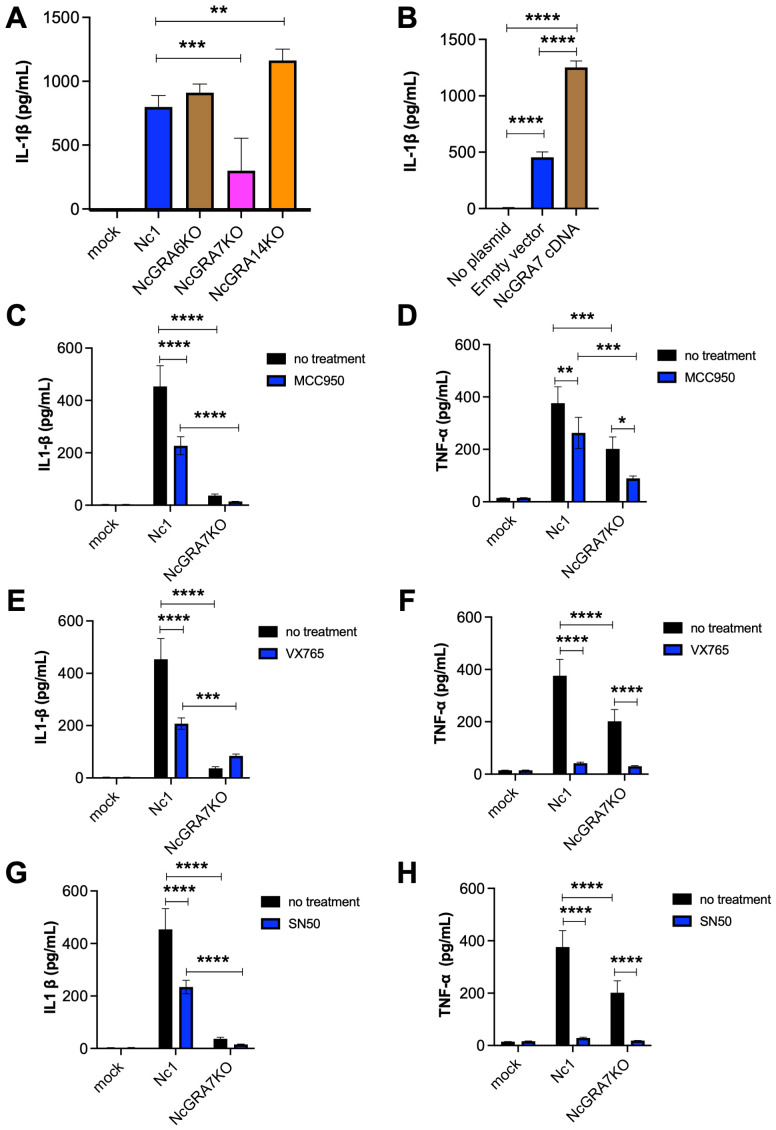
IL-1β and TNF-α production in THP-1 cells and inflammasome-reconstructed 293T cells. **(A)** THP-1 cells were infected with the parental strain Nc1 or the NcGRA6-, NcGRA7-, or NcGRA14-deficient (KO) parasites at a multiplicity of infection (MOI) of 2.5 or treated with medium only (mock). At 20 h postinfection, the culture supernatants were collected for analysis. **(B)** 293T cells were transfected with inflammasome-reconstruction plasmids encoding NLRP3, ASC, procaspase-1, and pro-IL-1β, together with NcGRA7 cDNA or an empty vector. Untransfected 293T cells were used as a negative control (no plasmid). At 20 h posttransfection, the culture supernatants were collected for analysis. **(C–H)** THP-1 cells were pretreated with 10 μM MCC950 (an NLARP3 inhibitor), 100 μM VX765 (a CASP1 inhibitor), and 18 μM SN50 (an NF-κB inhibitor) for 2 hr and then infected with the Nc1 strain of *N. caninum* at a MOI of 2.5 or treated with medium only (mock). At 20 h postinfection, the culture supernatants were collected for analysis. Each value represents the mean ± SD of 4 replicates (technical replicates) in one representative experiment. Each experiment (biological replicate) was repeated two **(G, H)**, three **(A, D, E, F)** and four times **(B, C)**. Statistically significant differences according to one-way ANOVA or two-way ANOVA and a Tukey–Kramer post hoc analysis (* *P* < 0.05, ** *P* < 0.01, *** *P* < 0.001, **** *P* < 0.0001).

Our previous study showed that *N. caninum* infection induces the production of inflammatory cytokines from mouse macrophages and that NcGRA6, NcGRA7, and NcGRA14 are involved in the activation of NF-κB signaling, calcium/calcineurin (NFAT) signaling, and cAMP/PKA (CRE) signaling (34). Therefore, we compared the effects of NcGRA6, NcGRA7 and NcGRA14 on the IL-1β production of THP-1 cells. As shown in **Figure 1A**, the level of IL-1β production induced by NcGRA7KO infection was significantly lower than that induced by infection with the parental strain Nc1 or other knockout lines. To confirm the effect of NcGRA7 on NLRP3 inflammasome activation, the NLRP3 inflammasome was reconstructed in Human embryonic kidney cells (293T cells) by transfection with plasmids encoding NLRP3, ASC, procaspase-1, and pro-IL-1β (35). Transfection with plasmids for reconstitution of the NLRP3 inflammasome stimulated IL-1β production in 293T cells, and additional transfection with NcGRA7 cDNA significantly enhanced IL-1β production (**Figure 1B**).

IL-1β production is regulated by two signaling pathways, the activation inflammasome and the NF-κB pathway. To evaluate the mechanism through which *N. caninum* infection regulates IL-1β, we tested MCC950 (an NLRP3 inhibitor), VX765 (a CASP1 inhibitor), and SN-50 (an NF-κB inhibitor) in THP-1 cells following infection with parental strain Nc1 and NcGRA7KO (**Figures 1C–H**). All inhibitors significantly suppressed IL-1β production induced by Nc-1 infection, whereas no inhibitory effect was observed in NcGRA7KO-infected cells (**Figures 1C, E, G**). Moreover, TNF-α production in NcGRA7KO-infected cells was markedly lower than that in Nc-1-infected cells (**Figures 1D, F, H**). Furthermore, treatment with any of the inhibitors led to a reduction in TNF-α production in both Nc-1- and NcGRA7KO-infected cells (**Figures 1D, F, H**). Western blot analysis of phospho–NF-κB p65 revealed that *N. caninum* infection induced phosphorylation of NF-κB p65; however, no significant difference in phosphorylation levels was observed between Nc-1-infected and NcGRA7KO-infected cells (**Supplementary Figure S1**). These findings suggest that NcGRA7 does not directly regulate NF-κB activation, but rather positively influences the production of IL-1β and TNF-α through post-transcriptional mechanisms, thereby leading to the upregulation of NLRP3.

### Effects of NcGRA7 on mitochondrial damage and apoptosis in THP-1 cells

**Figure 2:**
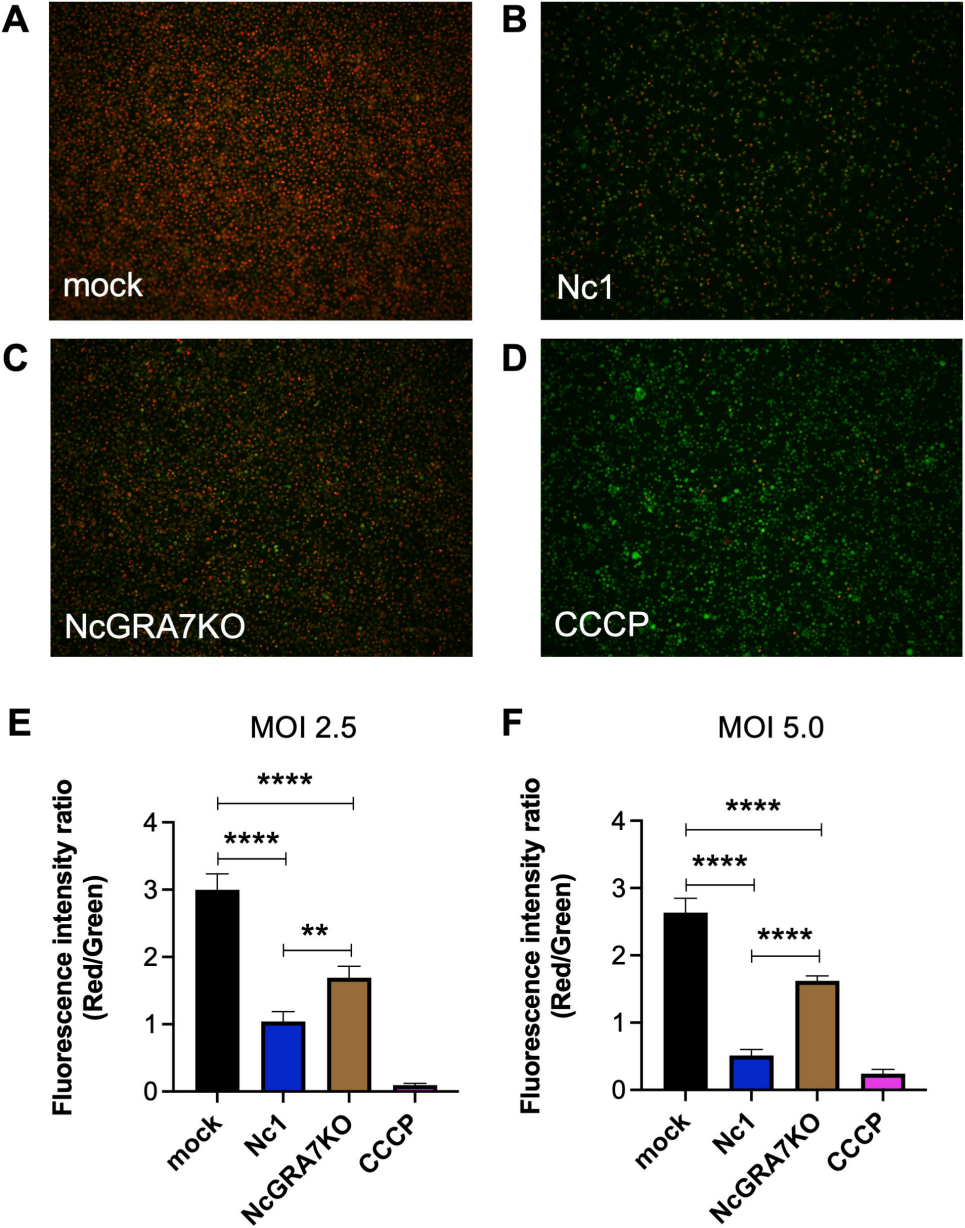
Quantification of the mitochondrial membrane potential (∆Ψm) in THP-1 cells. THP-1 cells were infected with mock **(A)**, the parental strain Nc1 **(B)**, NcGRA7-deficient (KO) parasites **(C)** at a multiplicity of infection (MOI) of 2.5 or treated with 100 μM CCCP **(D)**. At 20 h postinfection, the ∆Ψm in cells was measured using JC-1 staining, which allows a reduction in the red/green ratio (indicating depolarization) **(E)**. Each value represents the mean ± SD of 4 replicates (technical replicates) in one representative experiment. Each experiment (biological similar replicate) was repeated two times. **(F)** Results at a MOI of 5.0 are shown. Each value represents the mean ± SD of 4 replicates (technical replicates) in one representative experiment. Statistically significant differences according to one-way ANOVA or two-way ANOVA and a Tukey–Kramer post hoc analysis (** *P* < 0.01, **** *P* < 0.0001).

Recent studies have shown the pivotal roles of mitochondria in the initiation and regulation of the NLRP3 inflammasome, suggesting mitochondrial destabilization or damage. Therefore, we measured mitochondrial membrane potential (MtMP), a key indicator of mitochondrial activity, in *N. caninum*-infected THP-1 cells (**Figure 2**). *N. caninum* infection decreased MtMP levels, as shown by the control CCCP treatment which inhibits mitochondrial function by uncoupling for oxidative phosphorylation. Accordingly, we could observe the MtMP of NcGRA7KO-infected cells was significantly greater than that of parental Nc1 strain-infected cells at both a multiplicity of infection (MOI) of 2.5 and a MOI of 5.0.

**Figure 3:**
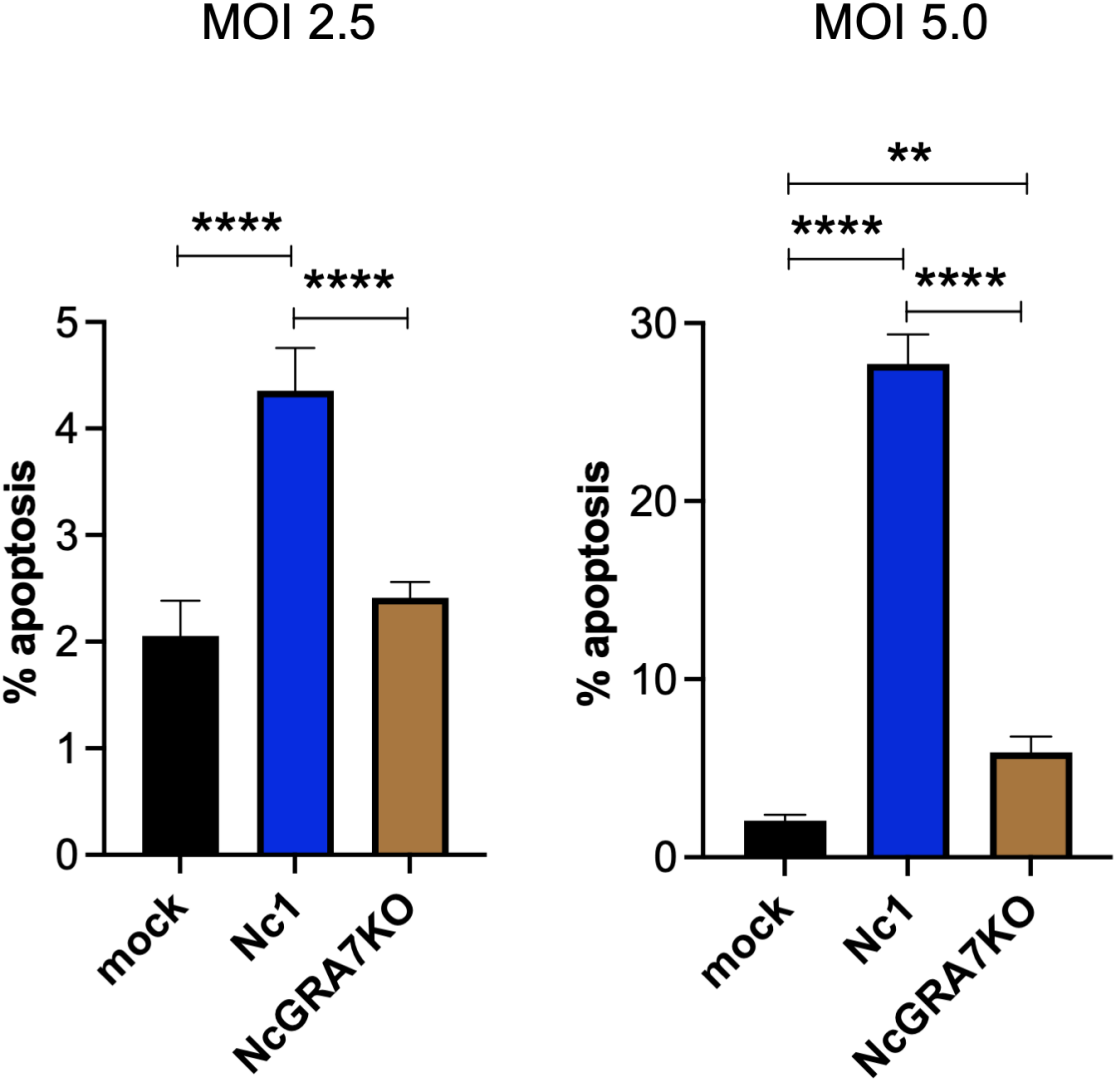
Quantification of THP-1 cell apoptosis. THP-1 cells were infected with mock, the parental strain Nc1, NcGRA7-deficient (KO) parasites at multiple infections (MOI) of 2.5 and 5.0. At 20 h postinfection, early apoptosis was measured using PE-conjugated Annexin V and 7-AAD. Each value represents the mean ± SD of 4 replicates (technical replicates) in one representative experiment. Each experiment (biological replicate) was repeated one (MOI of 2.5), two (MOI of 5) times. Statistically significant differences according to one-way ANOVA or two-way ANOVA and a Tukey–Kramer post hoc analysis (** *P* < 0.01, **** *P* < 0.0001).

Because mitochondrial damage induces apoptosis, we examined whether *N. caninum* infection induced the apoptosis of THP-1 cells (**Figure 3**). Compared with mock-infected THP-1 cells, *N. caninum* infection induced apoptosis in THP-1 cells at both a MOI of 2.5 and a MOI of 5.0. As expected, NcGRA7KO decreased host cell apoptosis.

### Identification of NcGRA7-binding proteins

**Figure 4:**
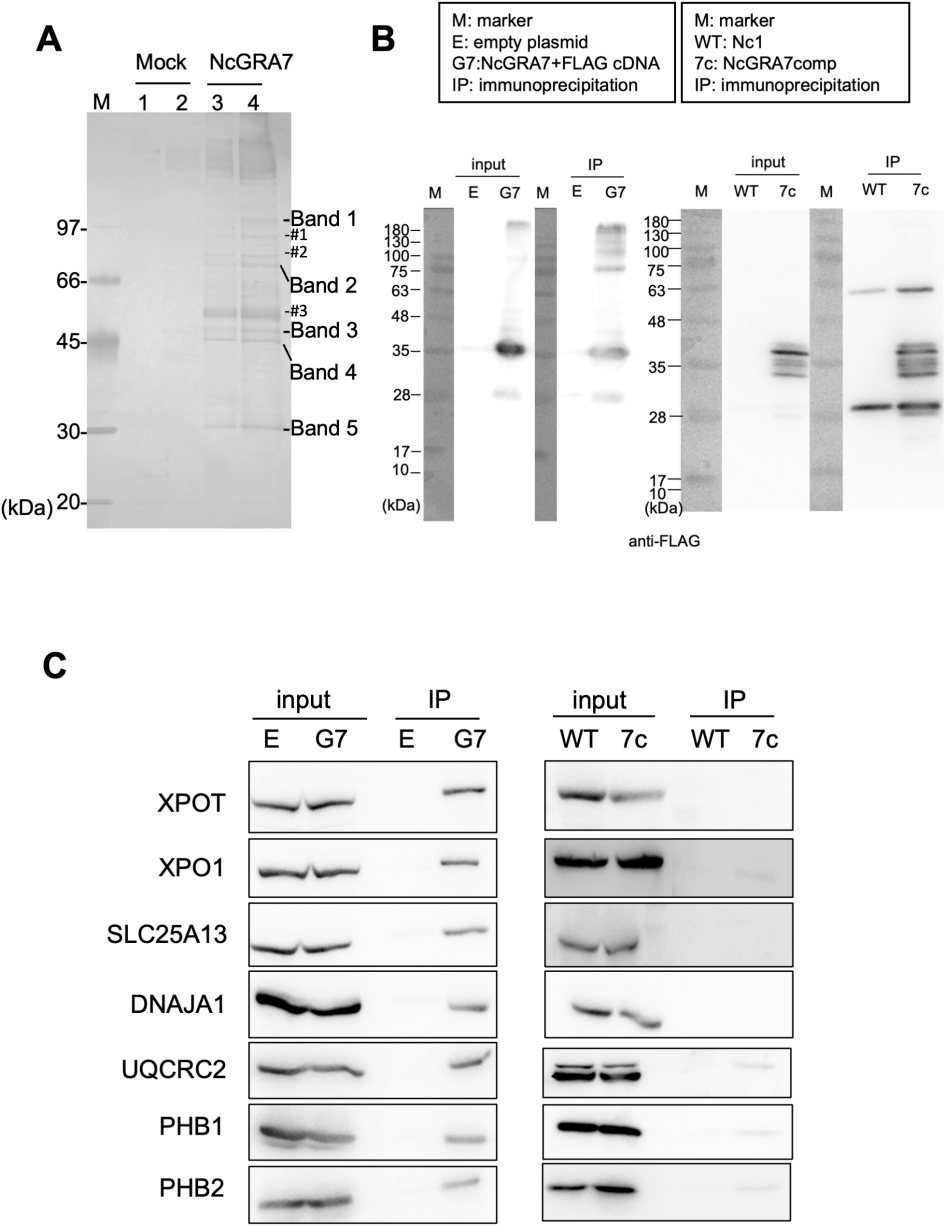
Characterization of NcGA7-binding proteins. **(A)** Silver staining for SDS–PAGE to identify NcGRA7-binding proteins by an anti-FLAG immunoprecipitation assay using 293T cells transfected with empty plasmid (Mock, Lanes 1 and 2) or NcGRA7 cDNA fused with a FLAG tag (Lanes 3 and 4). Enriched or exclusively detected proteins are shown as five bands, while the other three bands (#1, #2 and #3) were excluded from the MS analyses. M: molecular marker. Each experiment (biological replicate) was repeated two times. **(B, C)** Western blots of the fractions eluted from the anti-FLAG immunoprecipitate were analyzed by immunoblotting using antibodies against FLAG **(B)**, XPOT, XPO1, SLC25A13, DNAJA1, UQCRC2, PHB1, and PHB2 **(C)**. 293T cells transfected with empty plasmid **(E)** or NcGRA7 cDNA fused with a FLAG tag (G7) at 20 h posttransfection and HFFs infected with the parental strain Nc1 of *N. caninum* (WT) and NcGRA7-complemented (7c) parasites at 40 h postinfection were used. Each experiment (similar biological replicate) was repeated two time **(B, C)**.

To clarify the mechanism of IL-1β production induced by NcGRA7, we aimed to identify NcGRA7-binding proteins by an anti-FLAG immunoprecipitation assay using 293T cells transfected with NcGRA7 cDNA fused with a FLAG tag (**Figure 4A**). Five or more proteins were detected, while the other three bands (#1, #2 and #3) were excluded from further analyses because similar bands were observed in mock samples (#1 and #2), and #3 may be glycoproteins because a typical ladder pattern seen in proteins with added sugar chains (**Supplementary Figure S2**). The proteins in the excised gel band from the NcGRA7 IP eluate compared to those in the mock control IP eluate were subjected to mass spectrometric analysis. The proteins possibly bound to NcGRA7 are listed in **Supplementary Table S1**. Interestingly, a score>90 in the Mascot search suggested that NcGRA7-FLAG might be able to sort mitochondria or cytosol in host cells because proteins related to these organelles were detected via MS analysis.

**Figure 5:**
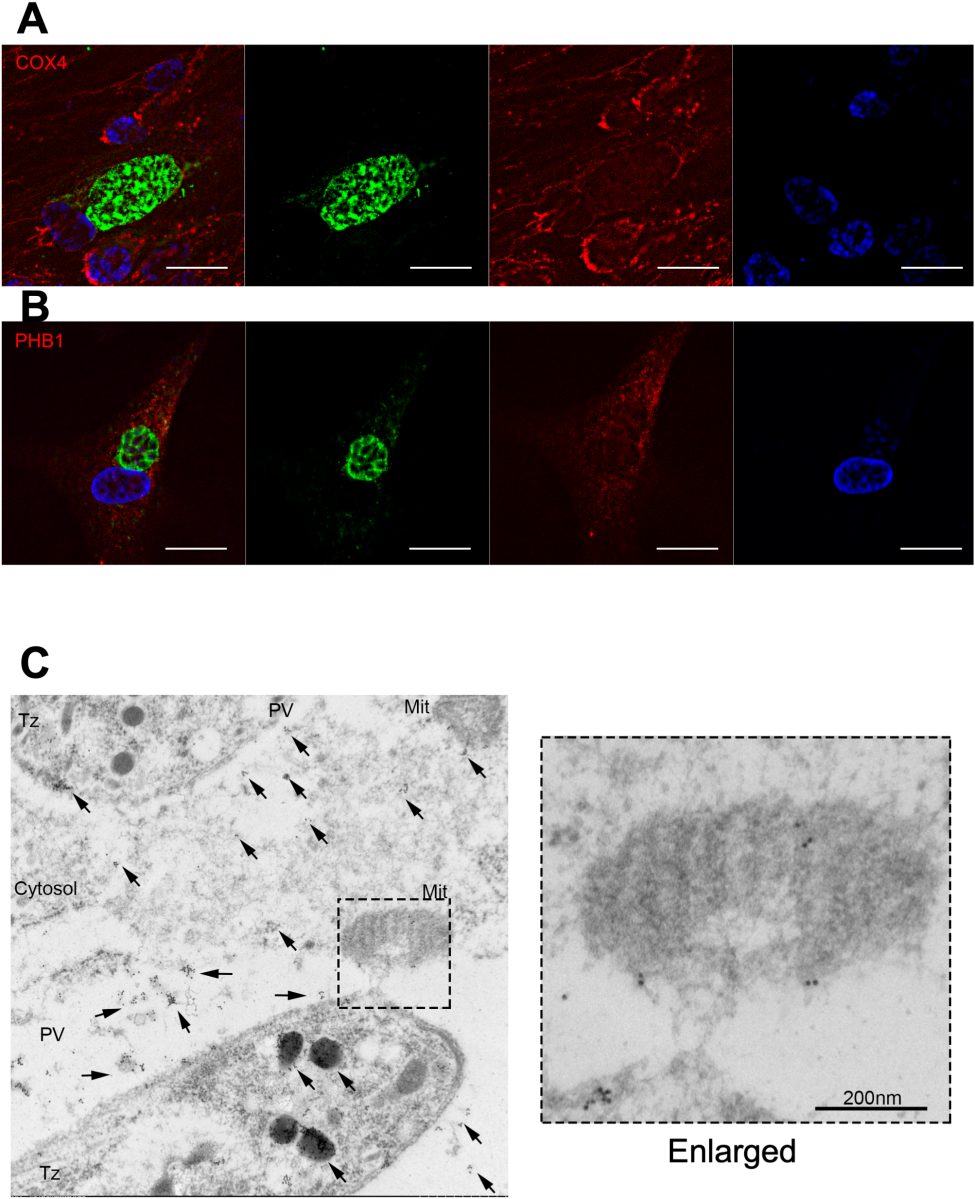
Indirect fluorescent antibody test of HFFs infected with the parental *N. caninum* strain Nc1. Indirect fluorescent antibody test of HFFs infected with the parental *N. caninum* strain Nc1 at 40 h postinfection using antibodies against CoxIV **(A)** and PHB1 **(B)**. The parasites were stained with an anti-NcGRA7 antibody. Green: NcGRA7. Red: CoxIV and PHB1. Blue: nuclear staining with Hoechst 33342. Each experiment (biological replicate) was repeated two times. **(C)** Immunoelectron microscopic analysis of intracellular *N. caninum* (Nc1 strain). Gold-labeled NcGRA7 (arrows) is distributed not only in the dense granules of a parasite and in the parasitophorous vacuolar space but also in the host cytoplasm and the host mitochondria. In the high-magnification field, gold-labeled NcGRA7 was localized to the cristae and the outer membrane of the mitochondria and to an approximately 100 nm structure with moderate electron density. DG, dense granule; PV, parasitophorous vacuole; Cytosol, host cytosol; Mit, mitochondria; Tz, Tachyzoite. Each experiment (biological replicate) was repeated two times.

Therefore, the fractions eluted from the anti-FLAG immunoprecipitate were analyzed by immunoblotting using antibodies against SLC25A13, DNAJA1, UQCRC2, PHB1 and PHB2, which are related to host mitochondria, and XPOT and XPO1, which localize to the nuclear envelope. As shown in **Figures 4B, C**, specific bands were observed in the NcGRA7 IP eluate prepared from 293T cells transfected with NcGRA7 cDNA but not in the mock control IP eluate. To confirm the physiological interaction of NcGRA7 with parasite-derived NcGRA7, eluted fractions from human foreskin fibroblasts (HFFs) infected with the wild-type Nc1 strain or the NcGRA7-complemented parasite, which expressed the NcGRA7 protein fused with a FLAG tag, were also analyzed. Among the tested antibodies, specific bands against XPO1, UQCRC2, PHB1 and PHB2 were detected (**Figure 4C**; **Supplementary Figure S3**). Then, we analyzed the colocalization of NcGRA7 with XPO1, UQCRC2 and PHB1 by IFAT (**Supplementary Figure S4**). Specific colocalization of NcGRA7 with XPO1 and UQCRC2 was not observed. On the other hand, NcGRA7 secreted from the PV was observed near host mitochondria, as determined by Cox IV, PHB1 (**Figure 5**) and MitoTracker (**Supplementary Figure S4D**) staining.

Immunoelectron microscopic analysis of intracellular parasites revealed that NcGRA7 was localized in dense granules and in the parasitophorous vacuolar space (**Figure 5C**). In the parasitophorous vacuolar space, NcGRA7 was diffusely distributed. In addition, NcGRA7 was also distributed in the host mitochondria and host cytoplasm. NcGRA7 was detected particularly in the cristae and outer membrane of mitochondria (high-magnification image of **Figure 5C**). NcGRA7 was also distributed within 50–100 nm structures, which exhibited moderate electron density, the host cytoplasm and the parasitophorous vacuolar space (high-magnification image of **Figure 5C**).

To confirm whether NcGRA7 affects mitochondrial morphology, we compared localization of host mitochondria marker Cox IV and PHB1 in HFFs infected with the wild-type Nc1 strain and NcGRA7KO parasites (**Supplementary Figure S5**). However, no significant morphological alterations were observed among the cells.

### Localization of NcGRA7 to the mitochondria and cytosol of host cells

**Figure 6:**
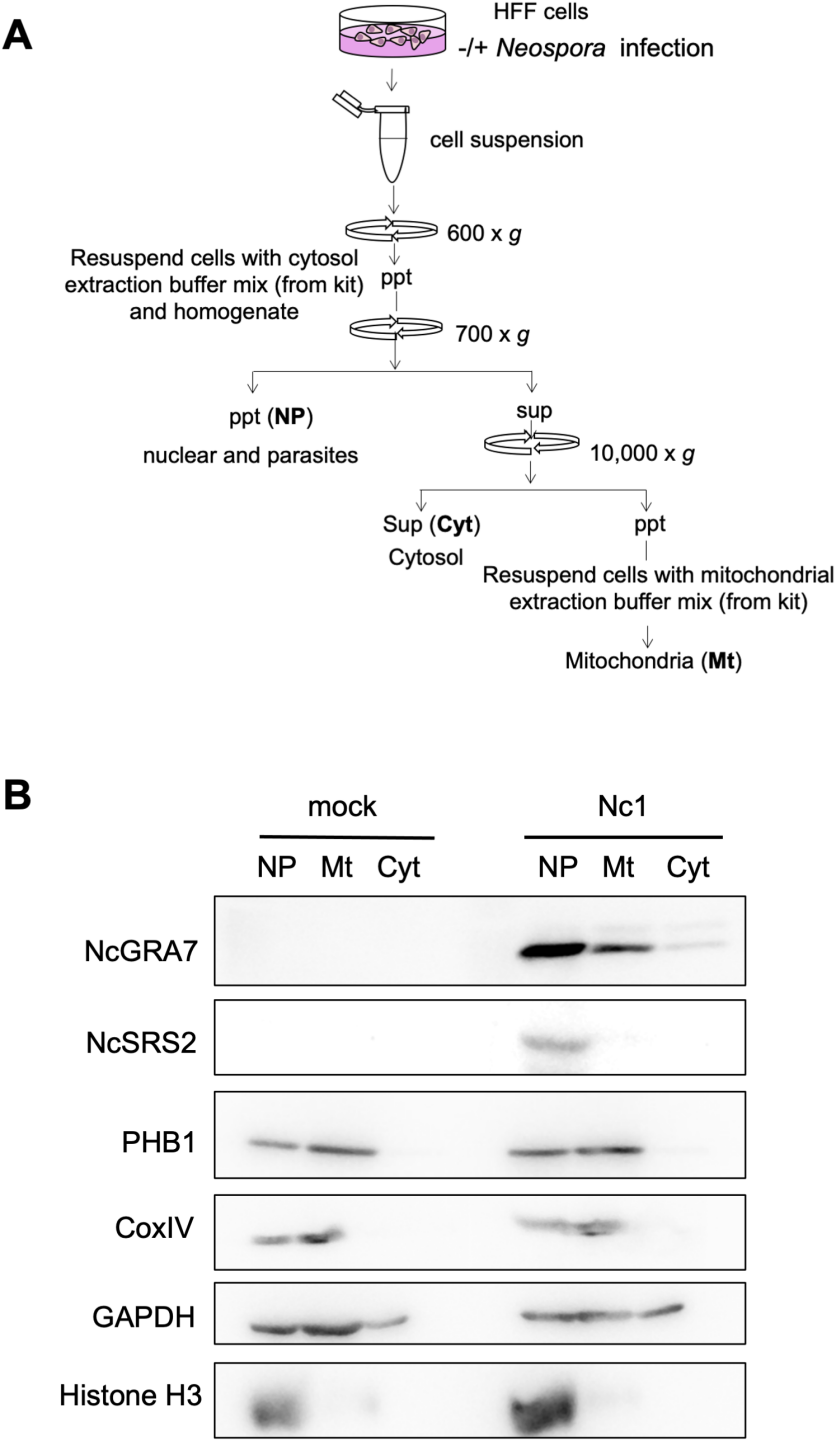
Western blot for the fractionation of cell homogenates. **(A)** Schematic view of the fractionation procedure used for the cell homogenates. HFFs infected with the parental Nc1 strain of *N. caninum* at 40 h postinfection and uninfected cells (mock) were homogenized, followed by sequential centrifugation to separate the nuclear/parasite (NP), mitochondrial (Mt) and cytosolic (Cyt) fractions. **(B)** Then, western blotting was performed using antibodies against NcGRA7, NcSRS2, PHB1, CoxIV, GAPDH, and Histone H3. M: molecular marker. Each experiment (similar biological replicate) was repeated three times.

To further confirm the localization of NcGRA7 in the cytosol and near mitochondria of host cells, we fractionated host cell extracts from *N. caninum*-infected cells and examined the mitochondrial and cytosolic localization of NcGRA7 using a biochemical approach (**Figure 6**). Nuclear protein Histone H3 and inner mitochondrial membrane (IMM) protein Cox IV were set as controls. As expected, endogenous NcGRA7 was detected in the fraction of mitochondria harboring PHB1 and in the cytosol of the host cells detected by GAPDH, which was distinct from the control of the parasite cell surface protein NcSRS2 (**Figure 6B**; **Supplementary Figure S6**).

**Figure 7:**
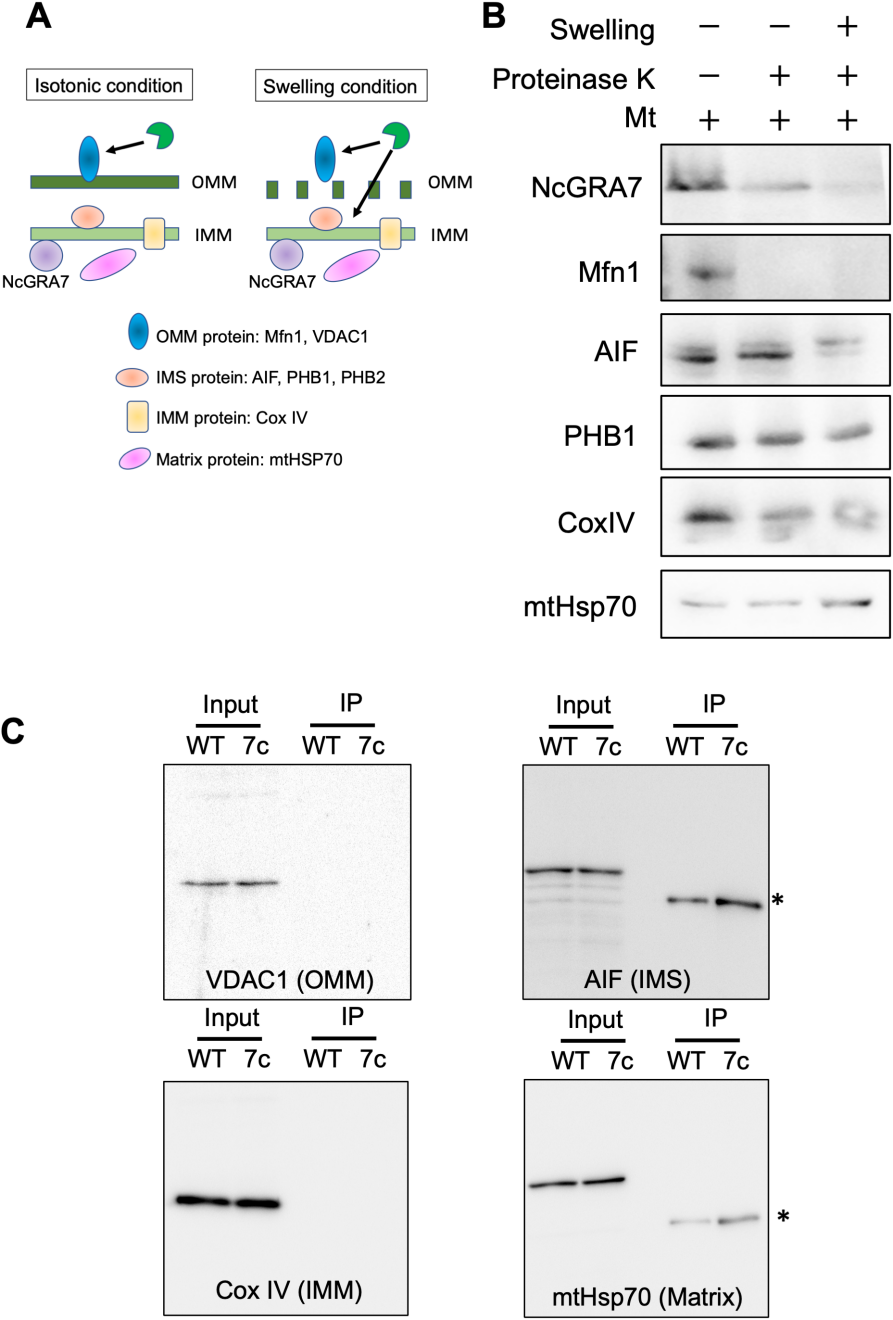
Localization of NcGRA7 in host mitochondria and binding to host mitochondrial proteins. **(A)** Schematic diagram of isolated mitochondria under isotonic and swelling conditions. Schematics of the isotonic (left) and swelling (right) conditions used in this study. Under isotonic conditions, proteinase K can access outer mitochondrial membrane (OMM) proteins but not the intermembrane space (IMS), inner mitochondrial membrane (IMM), or matrix proteins through membrane barriers. In addition, under swelling conditions, the protease can reach the IMS, followed by cleavage of both the OMM and IMS proteins. The inset shows mitochondrial proteins as markers for each category. **(B)** The mitochondrial fraction (Mt) isolated from HFFs was treated with proteinase K under isotonic (–) or hypotonic swelling (+) conditions. The reactants were developed by immunoblotting with antibodies against NcGRA7 or against several mitochondrial markers as indicated. OMM protein: Mfn1. IMS proteins: AIF, PHB1 and PHB2. IMM protein: COX IV. Matrix protein: mHsp70. Each experiment (biological replicate) was repeated two times. **(C)** Western blot of eluted fractions of HFFs infected with the parental strain Nc1 of *N. caninum* (WT) or NcGRA7-complemented parasites (7c) at 40 h post infection from anti-FLAG immunoprecipitation were analyzed by immunoblotting using antibodies against several mitochondrial markers as indicated. *Because mouse antibodies against FLAG, AIF and mHsp70 were used, the anti-mouse HRP secondary antibody was used to detect the anti-FLAG mouse antibody.

Next, to investigate the submitochondrial localization of NcGRA7, mitochondria isolated from *N. caninum*-infected cells were treated with proteinase K under isotonic conditions (**Figures 7A, B**; **Supplementary Figure S7**). NcGRA7 was partially digested by the protease under isotonic or swelling conditions, as observed for the control proteins for intermembrane space (IMS) or IMM, such as PHB1, COX IV and MtHSP70, indicating the distribution of NcGRA7 in the IMM to the matrix of host mitochondria. The interaction of NcGRA7 with other mitochondrial proteins was also examined by performing an anti-FLAG IP on eluted fractions from HFFs infected with the wild-type Nc1 strain or NcGRA7-complemented parasite (**Figure 7C**; **Supplementary Figure S8**). Immunoblotting using antibodies against VDAC1 (outer mitochondrial membrane (OMM) protein), AIF (IMS protein), Cox IV (IMM protein) and mtHsp70 (matrix protein) showed no binding with NcGRA7 and revealed the interaction of NcGRA7-FLAG with PHB1 and PHB2 (**Figure 4C**). PHBs form ring-like PHB complexes comprising multiple PHB1 and PHB2 subunits to maintain mitochondrial function (39). Together with the results shown in **Figure 4C**, these findings indicate that NcGRA7 may form a complex with PHB1 and PHB2.

### Effects of PHB1 on IL-1β and TNF-α production from *N. caninum*-infected THP-1 cells

**Figure 8:**
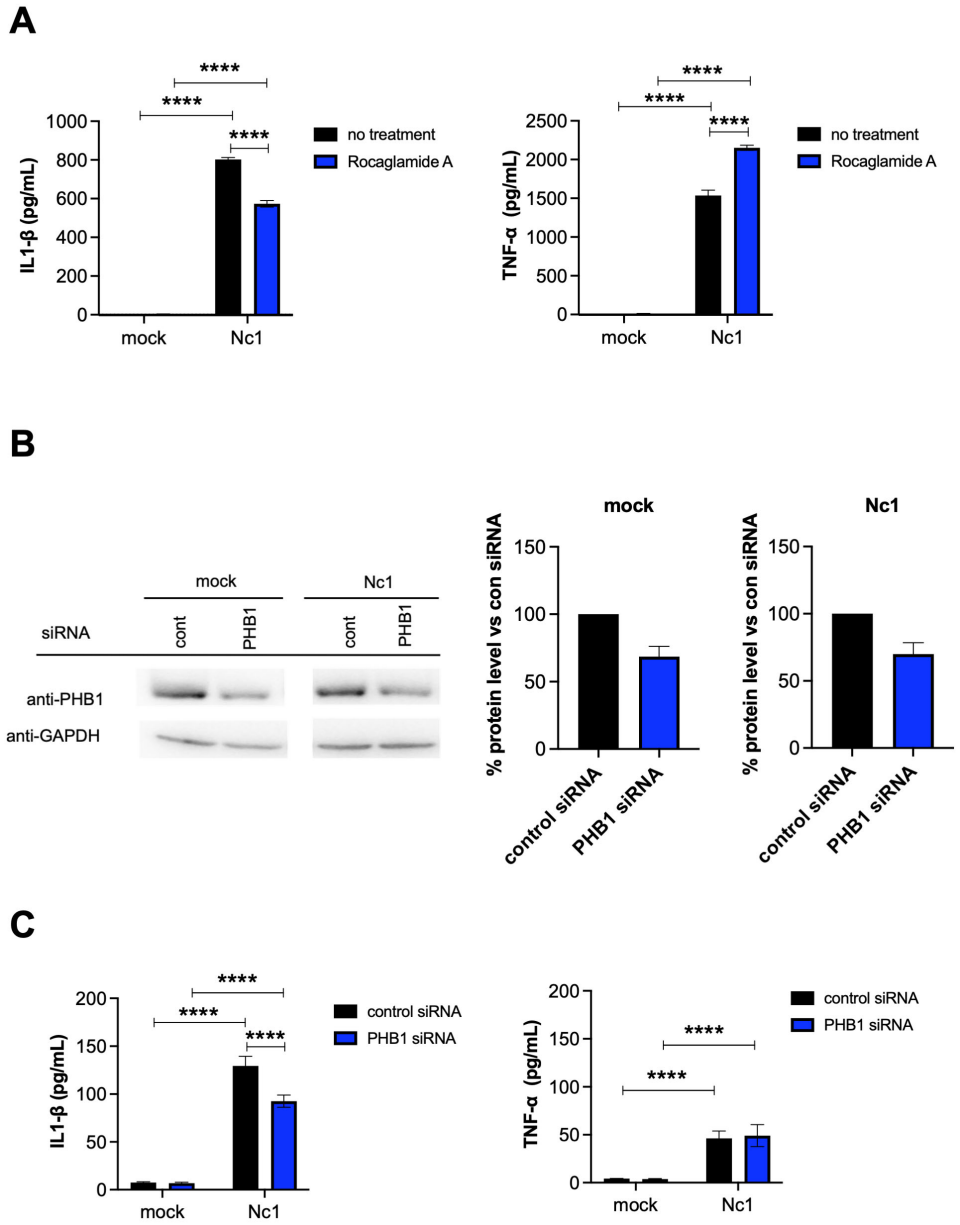
Effects of PHB1 on IL-1β and TNF-α production by THP-1 cells following *N. caninum* infection. **(A)** THP-1 cells were pretreated with 10 nM rocaglamide A (a PHB1 inhibitor), or medium only (no treatment) for 2 hr and then infected with the Nc1 strain of *N. caninum* at a multiplicity of infection (MOI) of 2.5 or treated with medium only (mock). At 20 h post infection, the IL-1β and TNF-α concentration in the culture supernatants was measured. Each value represents the mean ± SD of 4 replicates (technical replicates) in one representative experiment. Each experiment (biological replicate) was repeated five times. *statistically significant differences according to two-way ANOVA and a Tukey–Kramer *post hoc* analysis (*P* < 0.05). **(B)** THP-1 cells were transfected with control siRNA (cont), or PHB1 siRNA for 20 hr and then infected with the Nc1 strain of *N. caninum* at a MOI of 2.5 or treated with medium only (mock). At 20 h postinfection, the culture supernatants and cells were collected for cytokine ELISA and western blotting, respectively. The protein levels of PHB1 in the cell lysates were analyzed via western blotting. The protein levels were quantified based on band intensity. The protein levels of PHB1 were normalized to the protein levels of GAPDH, and then the reduction of PHB1 protein levels by PHB1 siRNA were calculated by relative to the control siRNA in the mock or the parasite infection. Each value of % protein level represents the mean ± SEM of three independent experiments (biological replicates). **(C)** At 20 h postinfection, the IL-1β and TNF-α concentration in the culture supernatants from **(B)** was measured. Each value represents the mean ± SD of 4 replicates (technical replicates) in one representative experiment. Each experiment (biological replicate) was repeated three times. Statistically significant differences according to one-way ANOVA or two-way ANOVA and a Tukey–Kramer post hoc analysis (**** *P* < 0.0001).

To examine the effects of PHB1 on IL-1β production by *N. caninum*-infected THP-1 cells, we tested the effects of PHB1 inhibitor (**Figure 8A**). Rocaglamide A, derived from medicinal plants, have been demonstrated to interact directly with PHB1 and thus inhibit the interaction of PHB1 with Raf-1, impeding Raf-1/ERK signaling cascades and significantly suppressing cancer cell metastasis (40). IL-1β production was significantly inhibited in *N. caninum*-infected THP-1 cells by treatment with Rocaglamide A while TNF-α production was increased (**Figure 8A**).

To further confirm the effects of PHB1 on IL-1β production following infection of THP-1 cells with *N. caninum*, siRNA-mediated knockdown of PHB1 was carried out (**Figure 8B**). Transfection of mock-infected cells with PHB1 siRNA decreased PHB1 protein level by 31.4% compared to the transfection of mock-infected cells with control siRNA. Additionally, transfection with PHB1 siRNA reduced PHB1 protein level by 30.0% compared with transfection with control siRNA in Nc-1-infected cells (**Supplementary Figure S9**). As shown in **Figure 8C**, the transfection of *N. caninum*-infected THP-1 cells with PHB1 siRNA significantly decreased IL-1β production but not TNF-α production, indicating interaction of NcGRA7 and PHB1 might have a role on NLRP3 inflammasome pathway.

## Discussion

The pathogenesis and host defense against protozoan infections are strongly affected by innate immunity. MyD88-dependent TLR signaling is essential for triggering an appropriate immune response against *N. caninum* (7–9, 41). Moreover, NLRs have also been recognized as crucial elements of the innate immune system because of their capacity to identify and eradicate intracellular parasites (10, 42). *N. caninum* can activate a variety of PRRs, including TLR2 (8), TLR3 (9), TLR11 (43, 44), NOD2 (45), Dectin-1 (6), and NLRP3 (31), in innate immune cells to trigger the host immune response. In this work, we describe a novel mechanism of *N. caninum* survival: *N. caninum* uses a functional secretory protein from the parasite, NcGRA7, to communicate with host mitochondria. In fact, the host can benefit from the defensive effects of the NLRP3 inflammasome in response to infection by a variety of bacteria, viruses, fungi, and parasites. For this reason, the increased expression levels of NLRP3 and IL-1β prompted us to investigate how NLRP3 inflammasome activation and IL-1β secretion are induced. Two signals are necessary for the NLRP3 inflammasome to become activated in macrophages: the first signal is produced by NF-κB activation, which causes the upregulation of pro-IL-18, pro-IL-1β, and NLRP3; different DAMPs or PAMPs supply the second signal (46, 47).

In a previous study, LPS pretreatment was shown to be necessary to trigger the release of IL-1β and IL-18 in bone marrow-derived macrophages (BMDMs) infected with *N. caninum* (31). However, in another study, *N. caninum* alone was able to stimulate IL-1β production in peritoneal macrophages (29). This discrepancy could be connected to the fundamental distinctions between peritoneal macrophages and BMDMs (48). Additionally, the crucial inflammasome components for IL-1β secretion were identified in *T. gondii*-infected BMDMs from mice lacking ASC, Nlrp1b, Nlrp3, or both Nlrp1b and Nlrp3, which demonstrated a strong correlation between the NLRP3 inflammasome and IL-1β production (49). Nevertheless, in addition to data on NLRP3, data from *T. gondii*-infected mice lacking NLRP1 also demonstrated the protective function of NLRP1 against *T. gondii* infection (27). In peritoneal macrophages infected with *N. caninum*, predominantly upregulated expression of NLRP3 but not NLRP1 was observed (29). Because we observed IL-1β production in THP-1 cells following *N. caninum* infection, our hypothesis was that NLRP3 is primarily responsible for detecting the presence of *N. caninum* infection. According to Mineo et al. (2010) (50), the excretory secretion antigen (ESA) of *N. caninum* tachyzoites can cause IL-1β production, indicating the potential of parasite-derived molecules for inflammasome activation. Importantly, we first found that NcGRA7 partially contributed to IL-1β production via the NLRP3 inflammasome.

NcGRA7 is a highly immunogenic dense granule protein in *N. caninum* (51) and is involved in the processes of cell invasion (52, 53). Additionally, our previous studies showed that NcGRA7 regulates the pathogenesis of neosporosis by modulating the host immune response (34), and a subunit vaccine based on recombinant NcGRA7 confers protective immunity against *N. caninum* infection in an infection model in mice and cattle (54, 55). Although NcGRA7 is an essential substance involved in the development of parasitophorous vacuoles (PVs) and the formation of the PV membrane (PVM), the secretion of NcGRA7 into the host cell cytosol was confirmed in *N. caninum*-infected cells (34), suggesting that NcGRA7 interacts with host molecules beyond the PVM. In fact, our MS analysis using immunoprecipitates of NcGRA7-FLAG detected interactions with XPO1, UQCRC2, PHB1 and PHB2. Importantly, IFAT, immunoelectron microscopy and biochemical analysis revealed that NcGRA7 was distributed in the IMM to the matrix of host mitochondria and formed a complex with PHB1 and PHB2. Although this is the first report on the association of *Neospora* protein with host mitochondria, further research is needed to determine the molecular mechanism involved in the trafficking of NcGRA7 beyond the PVM.

We hypothesized that the interaction of NcGRA7 with PHB1 and PHB2 in the inner mitochondrial membrane (IMM) likely activates the NLRP3 inflammasome and mediates IL-1β release in monocytes. The prohibitins PHB1 and PHB2 are highly conserved, multifunctional proteins present in eukaryotic nuclear and mitochondrial compartments. PHB1 is a tumor suppressor protein involved in cell cycle control (56). PHB1 has been found in mitochondria, the nucleus, and the plasma membrane, as well as in the extracellular space (57). PHB2 is a mitophagy receptor in the inner mitochondrial membrane that requires rupture of the outer mitochondrial membrane for interaction with the autophagosome-associated protein LC3 (58). In mitochondria, prohibitins mainly exist as membrane-bound ring complexes and function as chaperones, maintaining mitochondrial protein stability during protein synthesis and transportation (39, 59). Thus, NcGRA7 secreted from the PV may interact with prohibitins in the inner mitochondrial membrane after the rupture of the outer mitochondrial membrane because *N. caninum* infection causes mitochondrial damage. The activation of the NLRP3 inflammasome initiated by a variety of cellular signals, including K^+^ efflux, Ca^2+^ signaling, mitochondrial dysfunction, and lysosomal rupture, has recently been reported (14, 60, 61). Damaged mitochondria contain a variety of damage-associated molecular patterns, such as mitochondrial DNA, ROS, and N-formylated peptides, which are released into the cytoplasm in response to cell death and pathogen invasion to trigger inflammatory responses (62). In the present study, infection with the parental strain of *N. caninum* but not with NcGRA7KO decreased the mitochondrial membrane potential. These results suggested that NcGRA7 contributes to mitochondrial damage caused by *N. caninum* infection, leading to NLRP3 inflammasome activation.

According to some studies on protozoan infections, mice have an inhibited ability to clear *Leishmania* (63), *T. gondii* (27), and *Trypanosoma cruzi* (64, 65) and are more vulnerable to invasive pathogens if they lack NLRP3, ASC, and caspase-1/11 (or caspase-1) in mice, indicating the importance of NLRP3 inflammasome activation for parasite control. In the presence of inhibitors against caspase-1, pancaspase and NLRP3, *N. caninum*-infected peritoneal macrophages exhibit reduced IL-1β induction and fail to regulate parasite replication (29). It has been reported that in *N. caninum*-infected BMDMs from Nlrp3−/−, Asc−/−, and Caspase-1/11−/− mice, IL-1β maturation and caspase-1 cleavage are almost completely eliminated. Additionally, *in vivo*, NLRP3 inflammasome components are essential for *N. caninum* clearance, mouse survival, and Th1 response induction in response to *N. caninum* infection (31). One study revealed that caspase-1 inhibition in *N. caninum*-infected bovine macrophages resulted in more parasites being present in the parasitophorous vacuole, while ATP-induced inflammasome activation helped with parasite clearance. This finding suggested that the bovine inflammasome could be a potential target for future drug or vaccine development against *N. caninum* infection in cattle (30).

Another report revealed that early infection sites had high monocyte and neutrophil counts, although these sites are not able to restrict *N. caninum* reproduction. Taken together, these findings imply that the NLRP3 inflammasome is involved in the host response to *N. caninum* infection and plays a crucial role in restricting parasite development and potentially improving the Th1 response by stimulating IFN-γ production (31). In *T. gondii* and *N. caninum* infections, the recruitment of inflammatory monocytes to infection sites serves as essential for controlling parasite development and spread (7, 66). Innate immune responses at the early site of infection are crucial for protection against parasitic infections, and cytokines such as IFN-γ and IL-12 aid in parasite management. Monocytes/macrophages are triggered to migrate to the early site of infection by *N. caninum-*excreted and secreted antigens in a CCR5-dependent manner (8), and the majority of innate immune cells at the initial infection site are monocytes/macrophages and neutrophils during *N. caninum* infection (67). These two types of cells are crucial in the immune response to *N. caninum*, and mice with macrophage depletion are more susceptible to infection (68).

In this study, we demonstrated for the first time that NcGRA7 does not directly regulate NF-κB activation but rather positively modulates the production of IL-1β and TNF-α through post-transcriptional mechanisms, leading to the upregulation of NLRP3. It is plausible that inflammasome activation contributes to restricting the proliferation of intracellular *N. caninum*; however, it may also be associated with the pathogenesis of infection. A deeper understanding of the pivotal role of NLRP3 inflammasome activation in *N. caninum* infection could facilitate the development of novel therapeutic or vaccination strategies to combat this pathogen.

## Data Availability

The original contributions presented in the study are included in the article/**Supplementary Material**. Further inquiries can be directed to the corresponding author.
