# Supplementary material for: Mitochondrial damage and IL-1β production in monocytes caused by Neospora caninum infection are mediated by dense granule protein 7 and prohibitins
Source: Front Immunol. 2025 Nov 20;16:1408992. doi: 10.3389/fimmu.2025.1408992 (PMC12675473; doi:10.3389/fimmu.2025.1408992)
Supplement: Supplementary file 2 [file Table1.docx]

**Supplement materials**

**Supplement Figures**

**Fig. S1.** THP-1 cells were pretreated with 18 μM SN50 (an NF-κB inhibitor) for 2 hr and then infected with the parental strain Nc1 or the NcGRA7-deficient (KO) parasites of *N. caninum* at a MOI of 2.5 or treated with medium only (mock). At 4 h postinfection, the cells were collected for western blotting using antibodies against phospho-NF-κB p65 (pNFkB), NF-κB p65 and GAPDH. M: molecular marker.


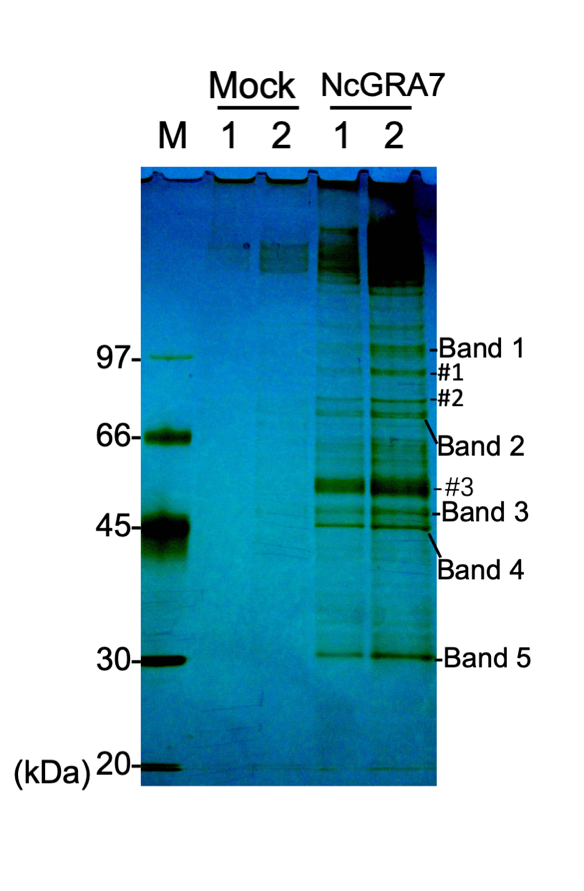


**Fig. S2.** High contrast image of silver staining for SDS‒PAGE to identify NcGRA7-binding proteins by an anti-FLAG immunoprecipitation assay using 293T cells transfected with empty plasmid (Mock, Lanes 1 and 2) or NcGRA7 cDNA fused with a FLAG tag (Lanes 3 and 4). Enriched or exclusively detected proteins are shown as five bands, while the other three bands (#1, #2 and #3) were excluded from the MS analyses. M: molecular marker. The bands (#1, #2) were seen in mock. Lanes 1 and 3: low loading volume, lanes 2 and 4: high loading volume.


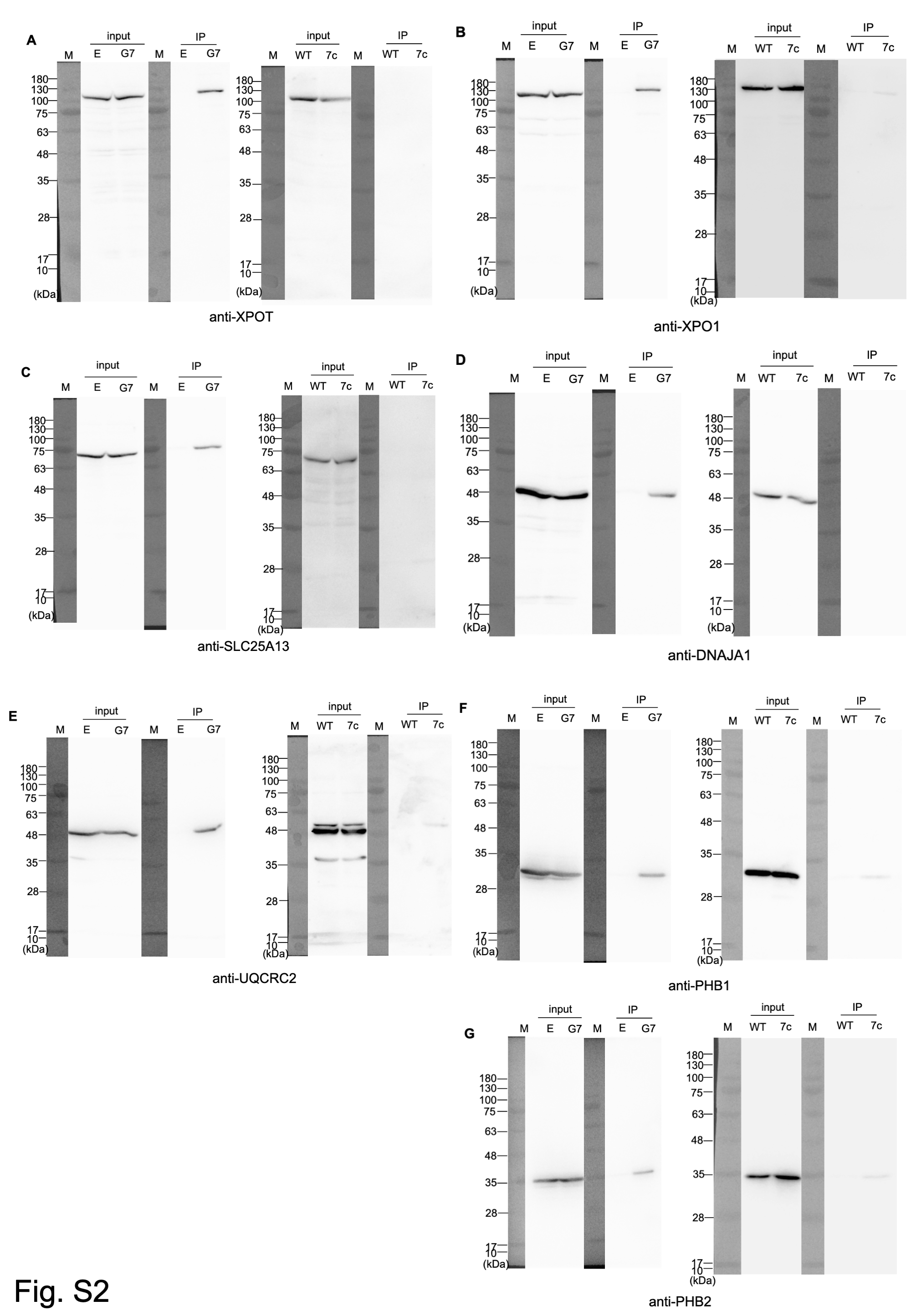


**Fig. S3.** Full images of Fig. 4C. Western blots of the fractions eluted from the anti-FLAG immunoprecipitate were analyzed by immunoblotting using antibodies against XPOT (A), XPO1 (B), SLC25A13 (C), DNAJA1 (D), UQCRC2 (E), PHB1 (F), and PHB2 (G). 293T cells transfected with empty plasmid (E) or NcGRA7 cDNA fused with a FLAG tag (G7) at 20 h posttransfection and HFFs infected with the parental strain Nc1 of *N. caninum* (WT) and NcGRA7-complemented (7c) parasites at 40 h postinfection were used.


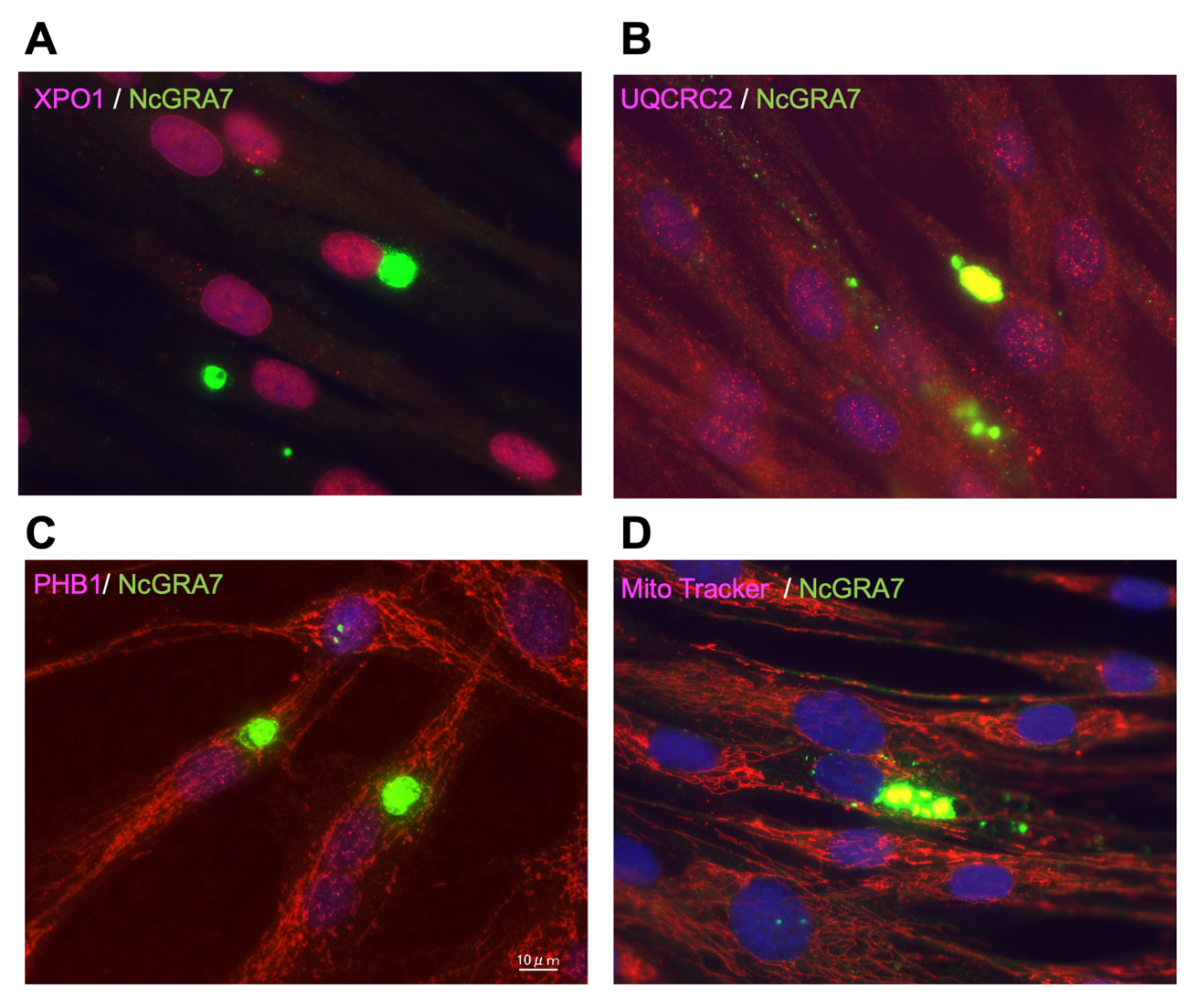


**Fig. S4.** Indirect fluorescent antibody test of HFFs infected with the parental *N. caninum* strain Nc1 at 40 h postinfection using anti-NcGRA7 mouse serum and antibodies against XPO1 (A), UQCRC2 (B) and PHB1 (C) or MitoTracker (D). Blue: nuclear staining with Hoechst 33342.

**Fig. S5.** Indirect fluorescent antibody test of HFFs infected with the parental strain Nc1 or the NcGRA7-deficient (KO) parasites of *N. caninum* at 40 h postinfection using antibodies against PHB1 and COX IV. Blue: nuclear staining with Hoechst 33342.


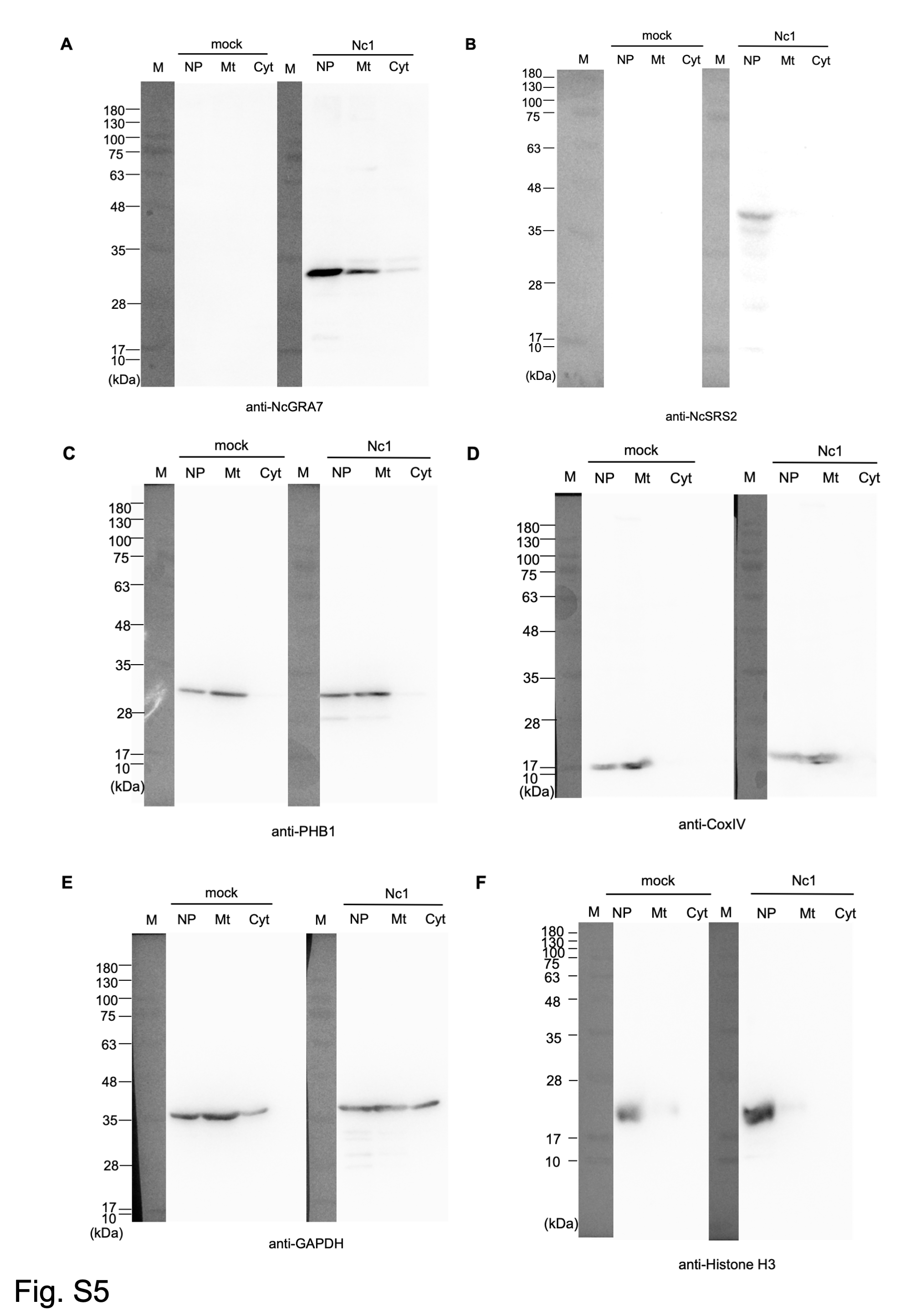


**Fig. S6.** Full images of Fig. 6B. HFFs infected with the parental Nc1 strain of *N. caninum* at 40 h postinfection and uninfected cells (mock) were homogenized, followed by sequential centrifugation to separate the nuclear/parasite (NP), mitochondrial (Mt) and cytosolic (Cyt) fractions. Then, western blotting was performed using antibodies against NcGRA7 (A), NcSRS2 (B), PHB1 (C), CoxIV (D), GAPDH (E) and Histone H3 (F). M: molecular marker.


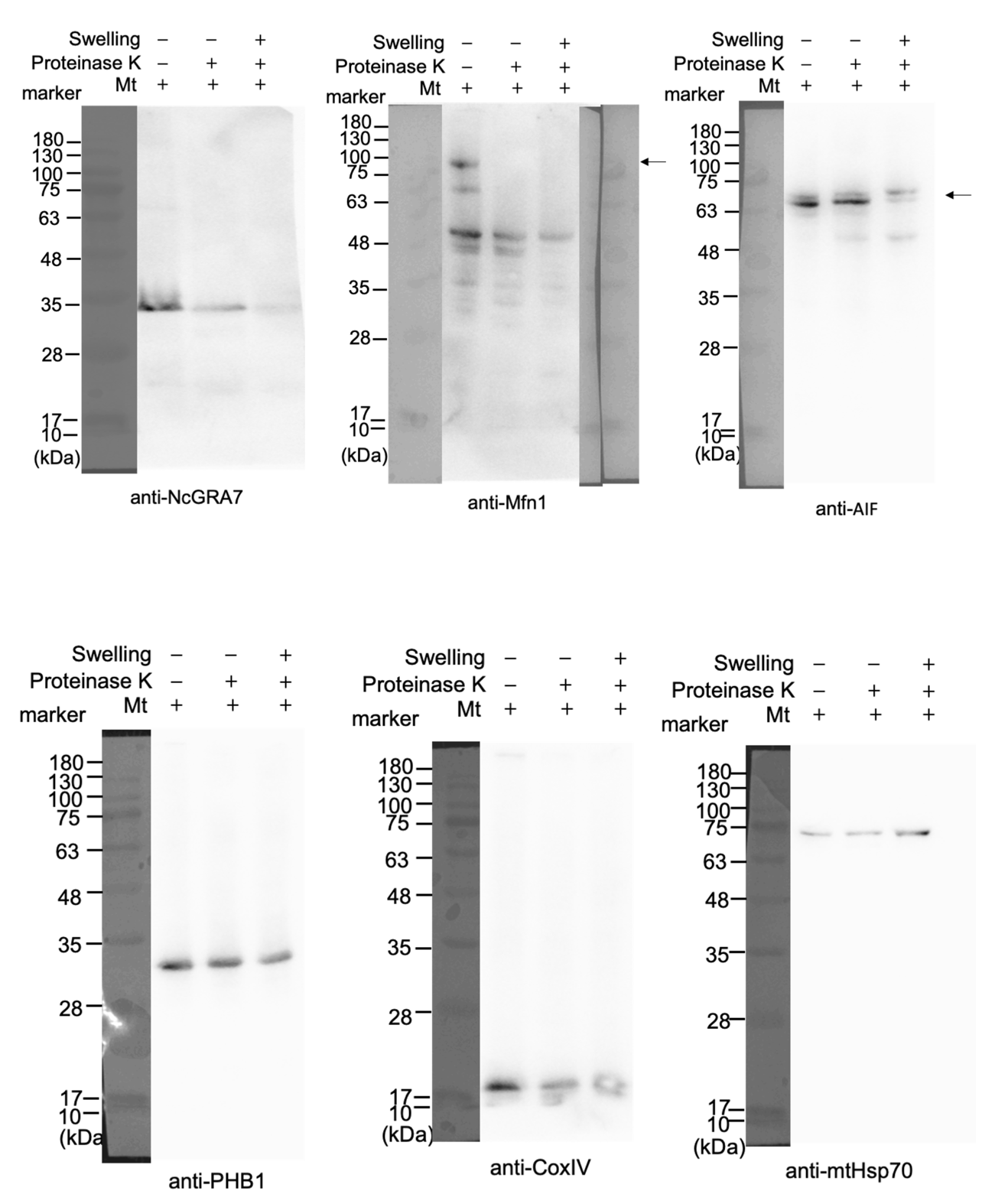


**Fig. S7.** Full images of Fig. 7B. The mitochondrial fraction (Mt) isolated from HFFs was treated with proteinase K under isotonic (-) or hypotonic swelling (+) conditions. The reactants were developed by immunoblotting with antibodies against NcGRA7 or against several mitochondrial markers as indicated. OMM protein: Mfn1. IMS proteins: AIF, PHB1 and PHB2. IMM protein: COX IV. Matrix protein: mHsp70.

**
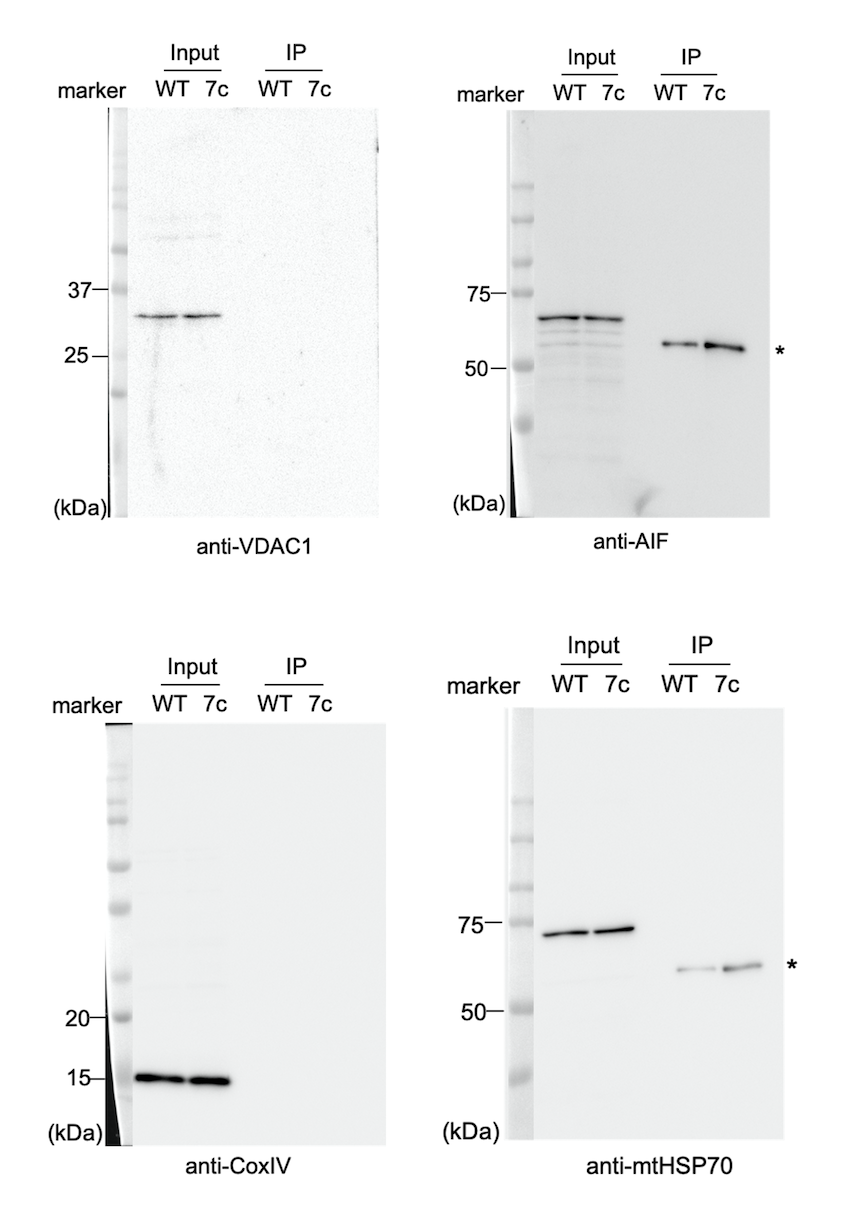
**

**Fig. S8.** Full images of Fig. 7C. Western blot of eluted fractions of HFFs infected with the parental strain Nc1 of *N. caninum* (WT) or NcGRA7-complemented parasites (7c) at 40 h post infection from anti-FLAG immunoprecipitation were analyzed by immunoblotting using antibodies against several mitochondrial markers as indicated. *Because mouse antibodies against FLAG, AIF and mHsp70 were used, the anti-HRP mouse secondary antibody was used to detect the anti-FLAG mouse antibody.


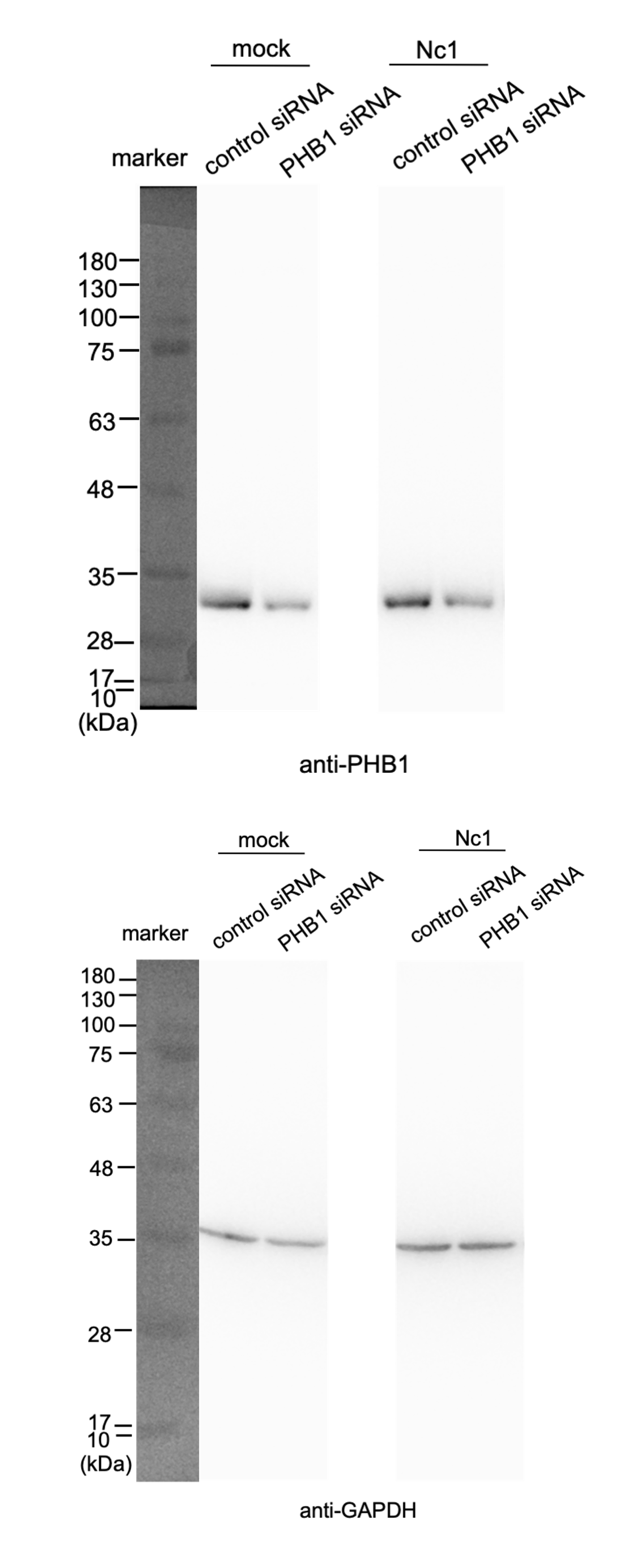


**Fig. S9.** Full images of Fig. 8B. THP-1 cells were transfected with control (cont), PHB1 siRNA for 20 hr and then infected with the Nc1 strain of *N. caninum* at a MOI of 2.5 or treated with medium only (mock). At 20 h postinfection, the culture supernatants and cells were collected for analysis. The protein expression levels of PHB1 in the cell lysates were analyzed via western blotting. The expression levels were quantified based on band intensity. The expression of each protein was normalized to the expression level of GAPDH, after which the expression of the target protein in each sample was calculated relative to that in mock-infected cells treated with control siRNA.
